# Supplementary material for: Central role of Tim17 in mitochondrial presequence protein translocation
Source: Nature. 2023 Aug 1;621(7979):627–34. doi: 10.1038/s41586-023-06477-8 (PMC10511324; doi:10.1038/s41586-023-06477-8)
Supplement: Supplementary file 1 — Supplementary Fig. 1, Tables 1–4 and additional references. [file 41586_2023_6477_MOESM1_ESM.pdf]

nature portfolio

https://doi.org/10.1038/s41586-023-06477-8

Supplementary information

Central role of Tim17 in mitochondrial presequence protein translocation

In the format provided by the authors and unedited

Supplementary Information

Central role of Tim17 in mitochondrial presequence protein translocation

Fielden et al.

Table of contents:

Supplementary Figure 1. Uncropped versions of gels/western blots of this study
Supplementary Table 1. Yeast strains used in this study
Supplementary Table 2. Plasmids used in this study
Supplementary Table 3. Primers used in this study
Supplementary Table 4. Antibodies used in this study

References:

14. Ieva, R. *et al.* Mgr2 functions as lateral gatekeeper for preprotein sorting in the mitochondrial inner membrane. *Mol. Cell* **56**, 641–652 (2014).
20. Gomkale, R. *et al.* Mapping protein interactions in the active TOM-TIM23 supercomplex. *Nat. Commun.* **12**, 5715 (2021).
21. Chacinska, A. *et al.* Mitochondrial presequence translocase: switching between TOM tethering and motor recruitment involves Tim21 and Tim17. *Cell* **120**, 817–829 (2005).
48. Gebert, M. *et al.* Mgr2 promotes coupling of the mitochondrial presequence translocase to partner complexes. *J. Cell Biol.* **197**, 595–604 (2012).
56. Qiu, J. *et al.* Coupling of Mitochondrial Import and Export Translocases by Receptor-Mediated Supercomplex Formation. *Cell* **154**, 596–608 (2013).
57. Longtine, M. S. *et al.* Additional modules for versatile and economical PCR-based gene deletion and modification in Saccharomyces cerevisiae. *Yeast* **14**, 953–961 (1998).
59. Chacinska, A. *et al.* Distinct Forms of Mitochondrial TOM-TIM Supercomplexes Define Signal-Dependent States of Preprotein Sorting. *Mol. Cell. Biol.* **30**, 307–318 (2010).
63. Voos, W. *et al.* Mitochondrial GrpE is present in a complex with hsp70 and preproteins in transit across membranes. *Mol. Cell. Biol.* **14**, 6627–6634 (1994).
64. Janke, C. *et al.* A versatile toolbox for PCR-based tagging of yeast genes: new fluorescent proteins, more markers and promoter substitution cassettes. *Yeast* **21**, 947–962 (2004).
65. Morgenstern, M. *et al.* Definition of a High-Confidence Mitochondrial Proteome at Quantitative Scale. *Cell Rep.* **19**, 2836–2852 (2017).
66. Priesnitz, C. *et al.* Coupling to Pam16 differentially controls the dual role of Pam18 in protein import and respiratory chain formation. *Cell Rep.* **39**, 110619 (2022).
67. Böttinger, L. *et al.* A complex of Cox4 and mitochondrial Hsp70 plays an important role in the assembly of the cytochrome *c* oxidase. *Mol. Biol. Cell* **24**, 2609–2619 (2013).
68. Vögtle, F. N. *et al.* Mutations in PMPCB Encoding the Catalytic Subunit of the Mitochondrial Presequence Protease Cause Neurodegeneration in Early Childhood. *Am. J. Hum. Genet.* **102**, 557–573 (2018).
69. Rampelt, H. *et al.* The mitochondrial carrier pathway transports non-canonical substrates with an odd number of transmembrane segments. *BMC Biol.* **18**, 2 (2020).
70. Weinhäupl, K. *et al.* Structural Basis of Membrane Protein Chaperoning through the Mitochondrial Intermembrane Space. *Cell* **175**, 1365-1379.e25 (2018).

Supplementary Figure 1. Uncropped versions of gels/western blots of this study

Figure 1c

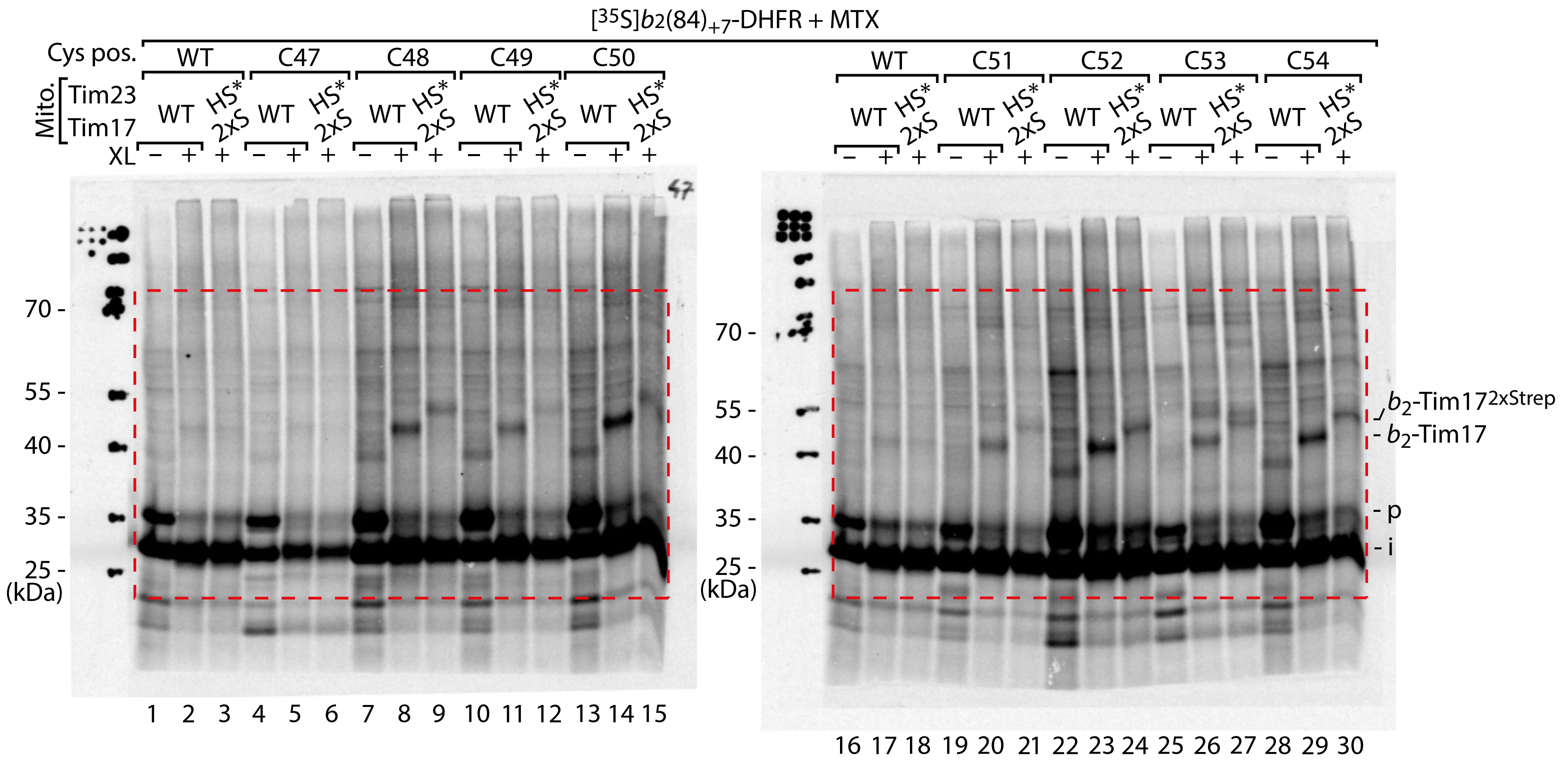

Figure 1d

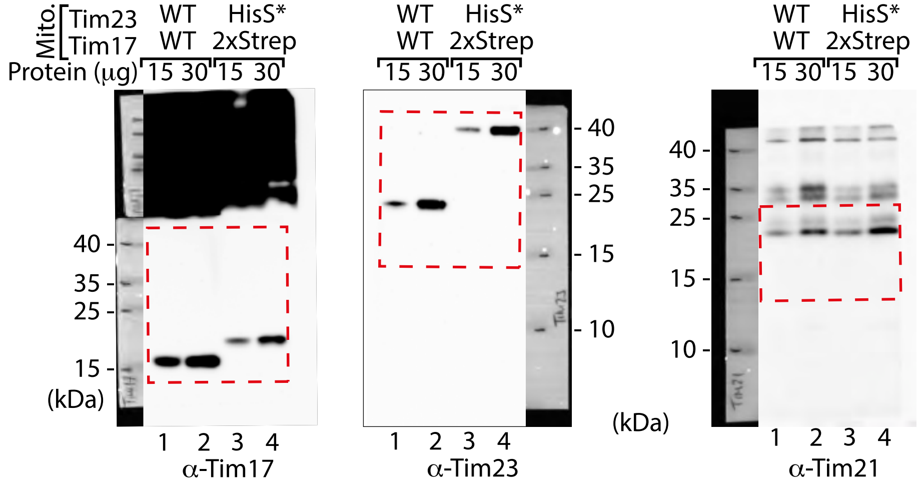

Figure 1f

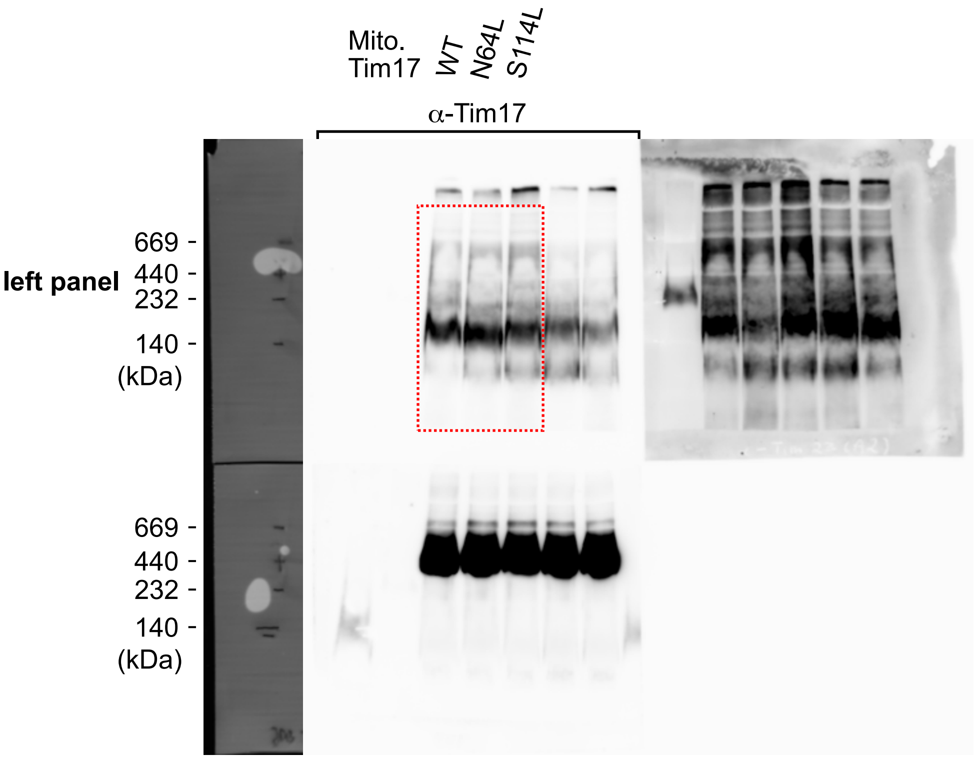

Mito.
Tim17
WT
N64L
S114L
α-Tim17
left panel
669 -
440 -
232 -
140 -
(kDa)
669 -
440 -
232 -
140 -
(kDa)

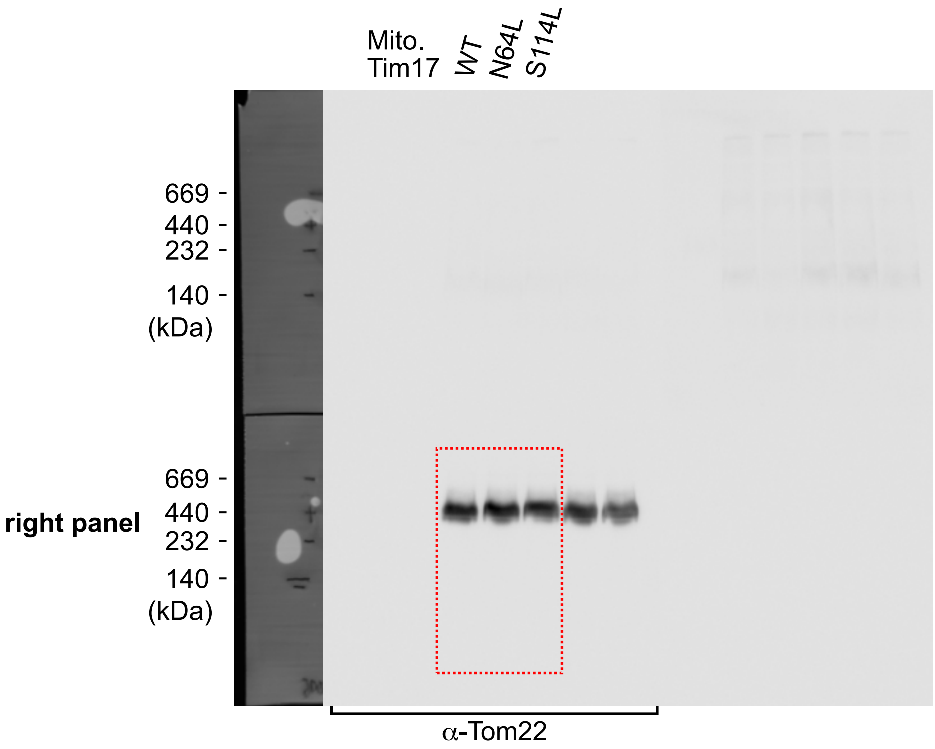

Mito.
Tim17
WT
N64L
S114L
right panel
669 -
440 -
232 -
140 -
(kDa)
669 -
440 -
232 -
140 -
(kDa)
α-Tom22

Figure 1g

left panel

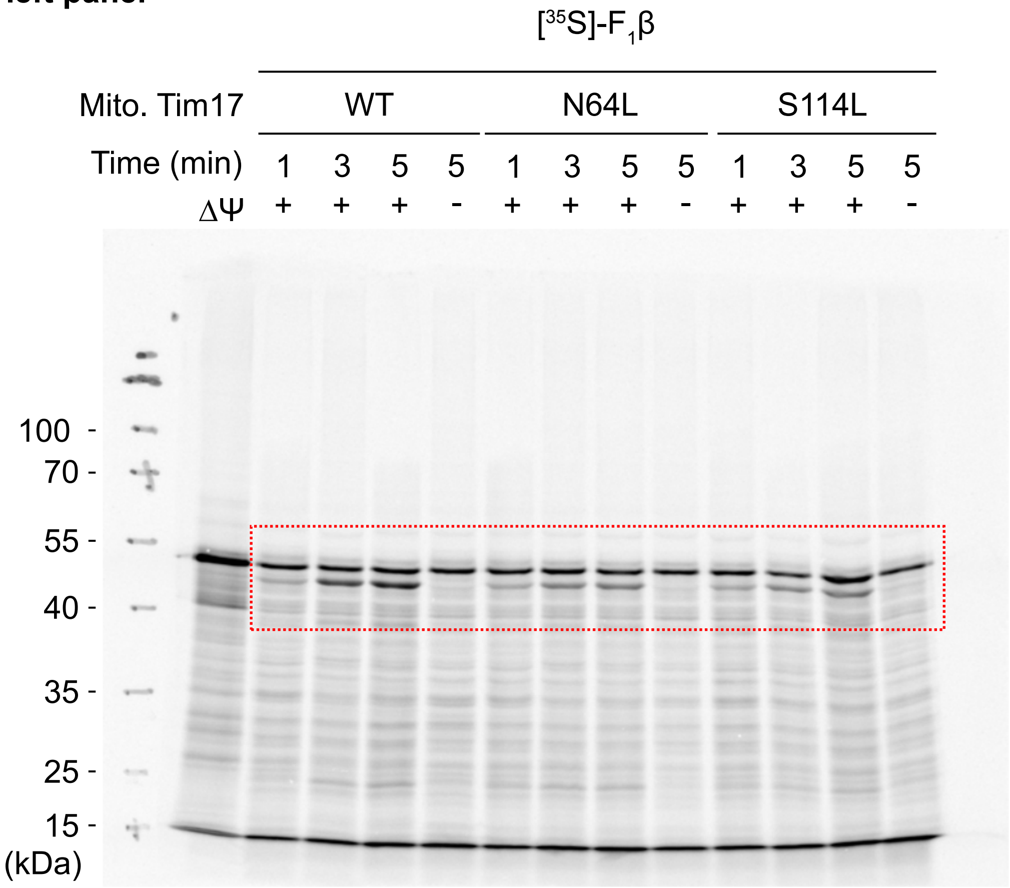

right panel

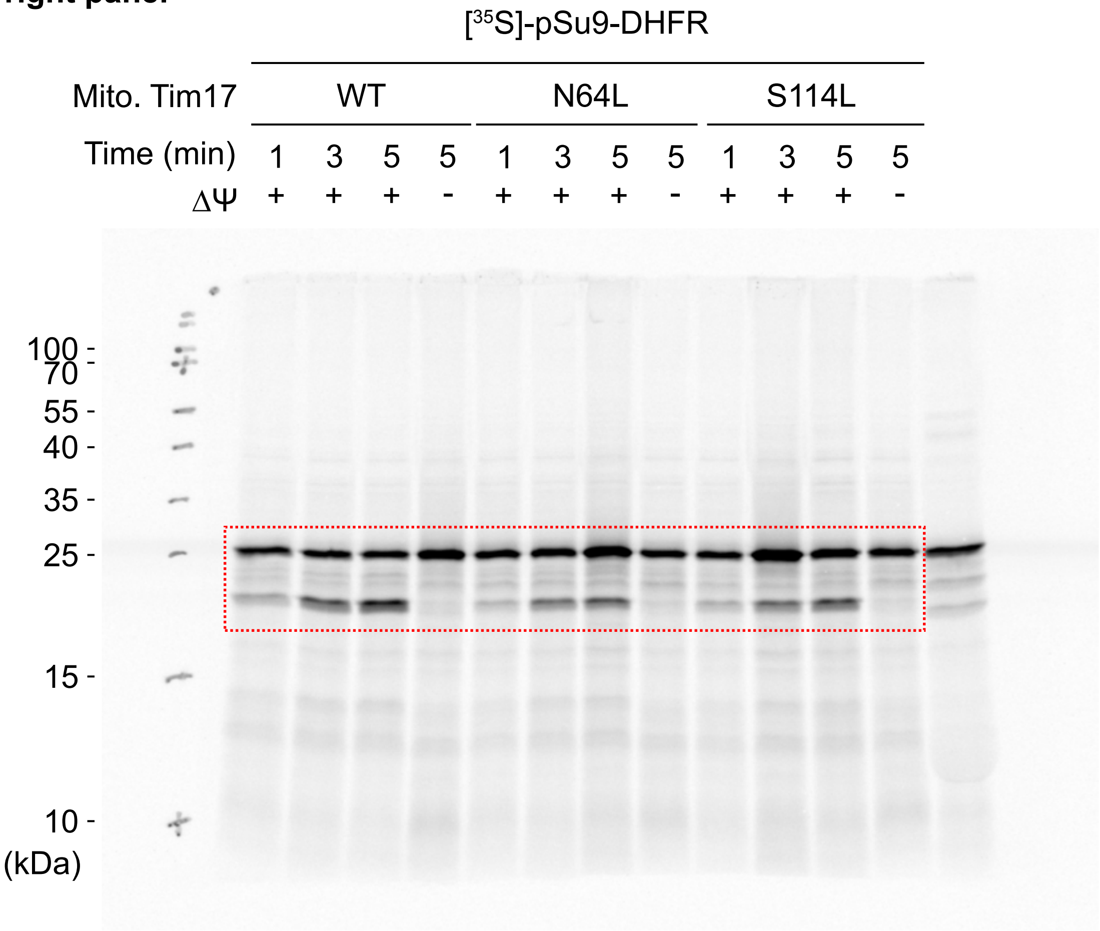

Figure 2b

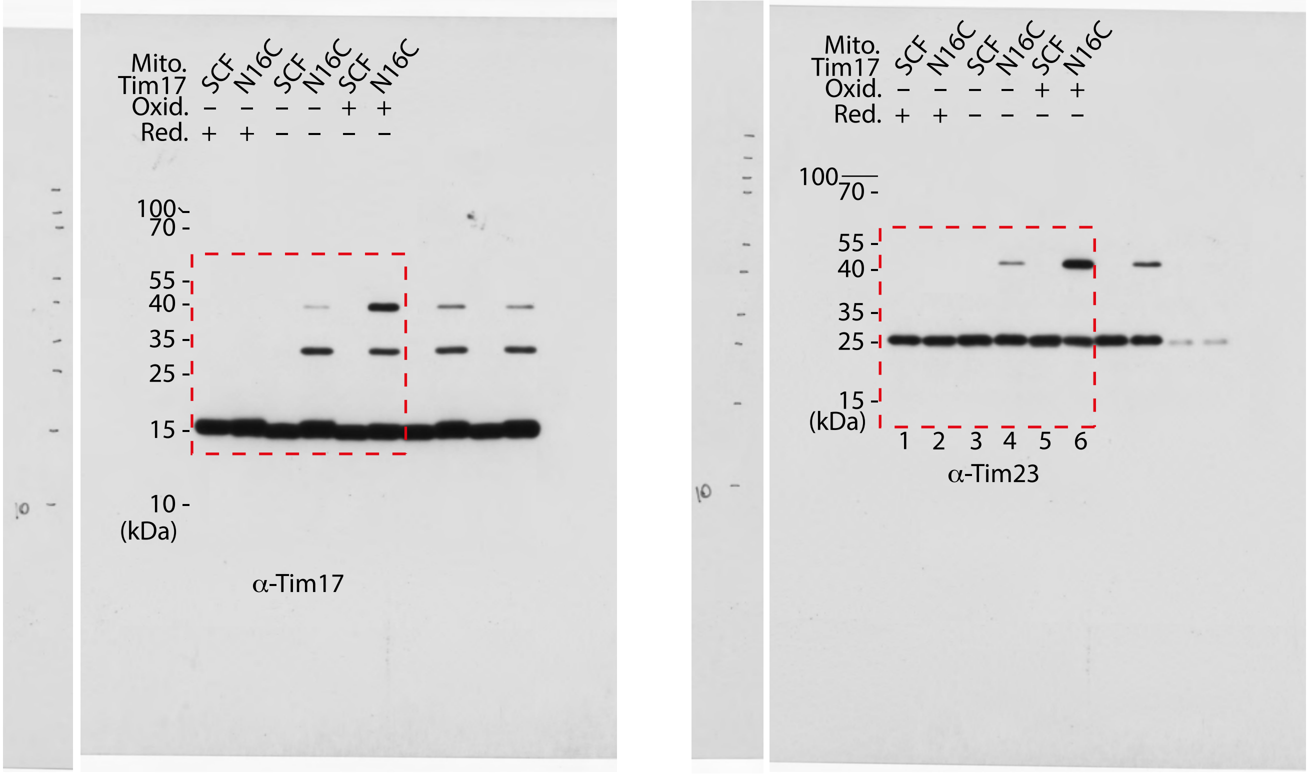

Figure 2c

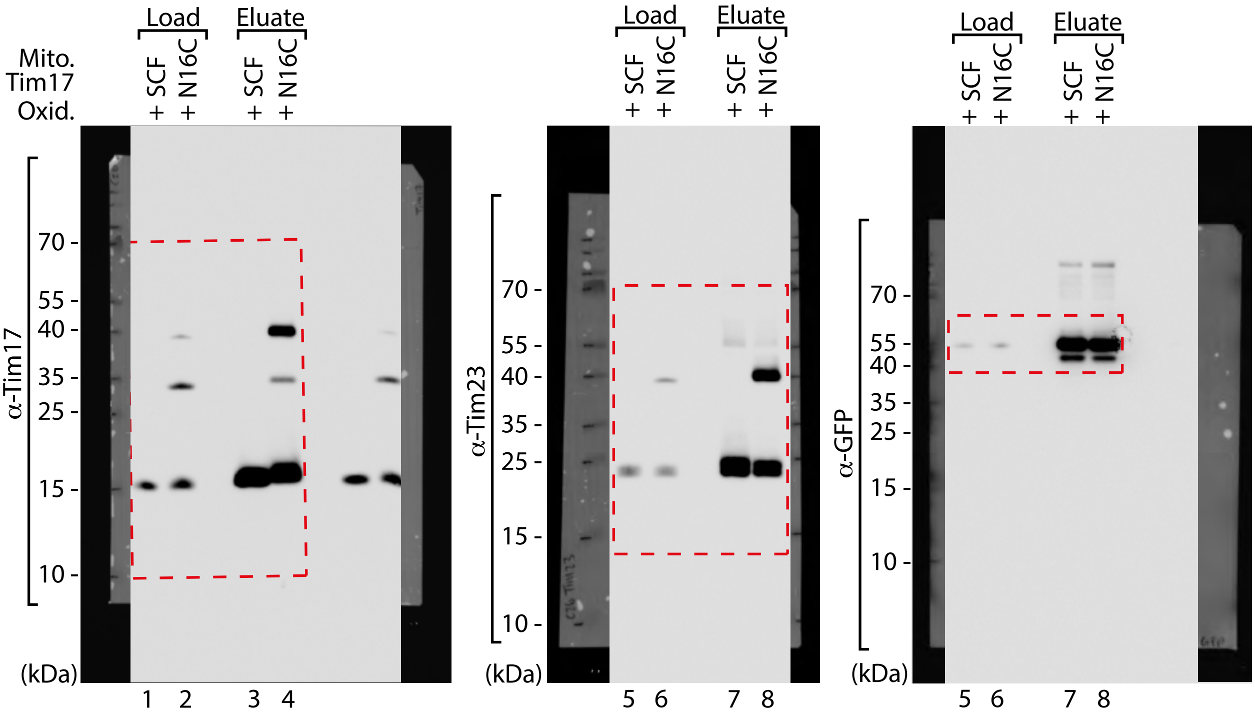

Figure 2d

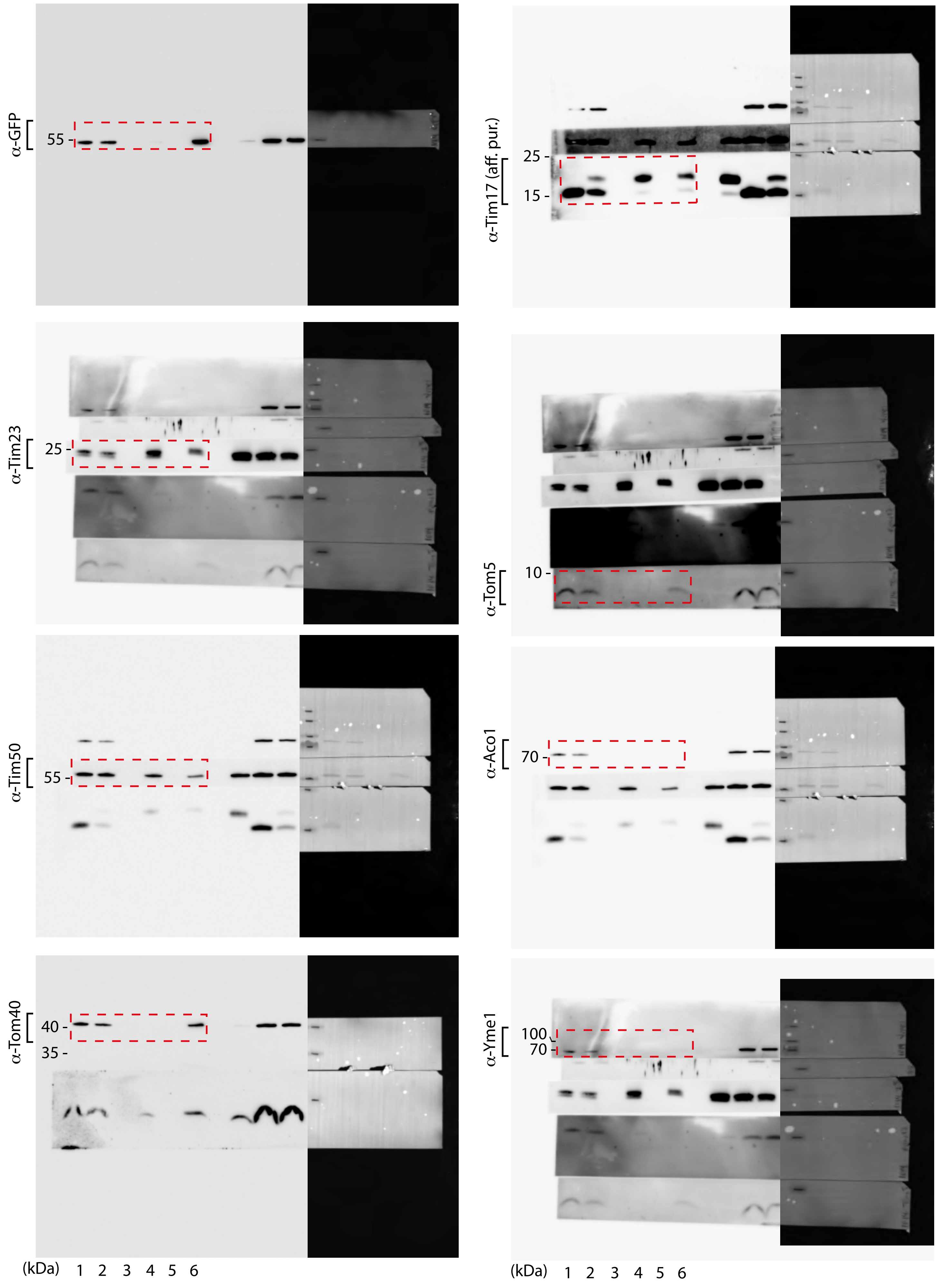

α-GFP
55 -
α-Tim17 (aff. pur.)
25 -
15 -
α-Tim23
25 -
α-Tom5
10 -
α-Tim50
55 -
α-Aco1
70 -
α-Tom40
40 -
35 -
α-Yme1
100
70 -
(kDa) 1 2 3 4 5 6
(kDa) 1 2 3 4 5 6

Figure 2e

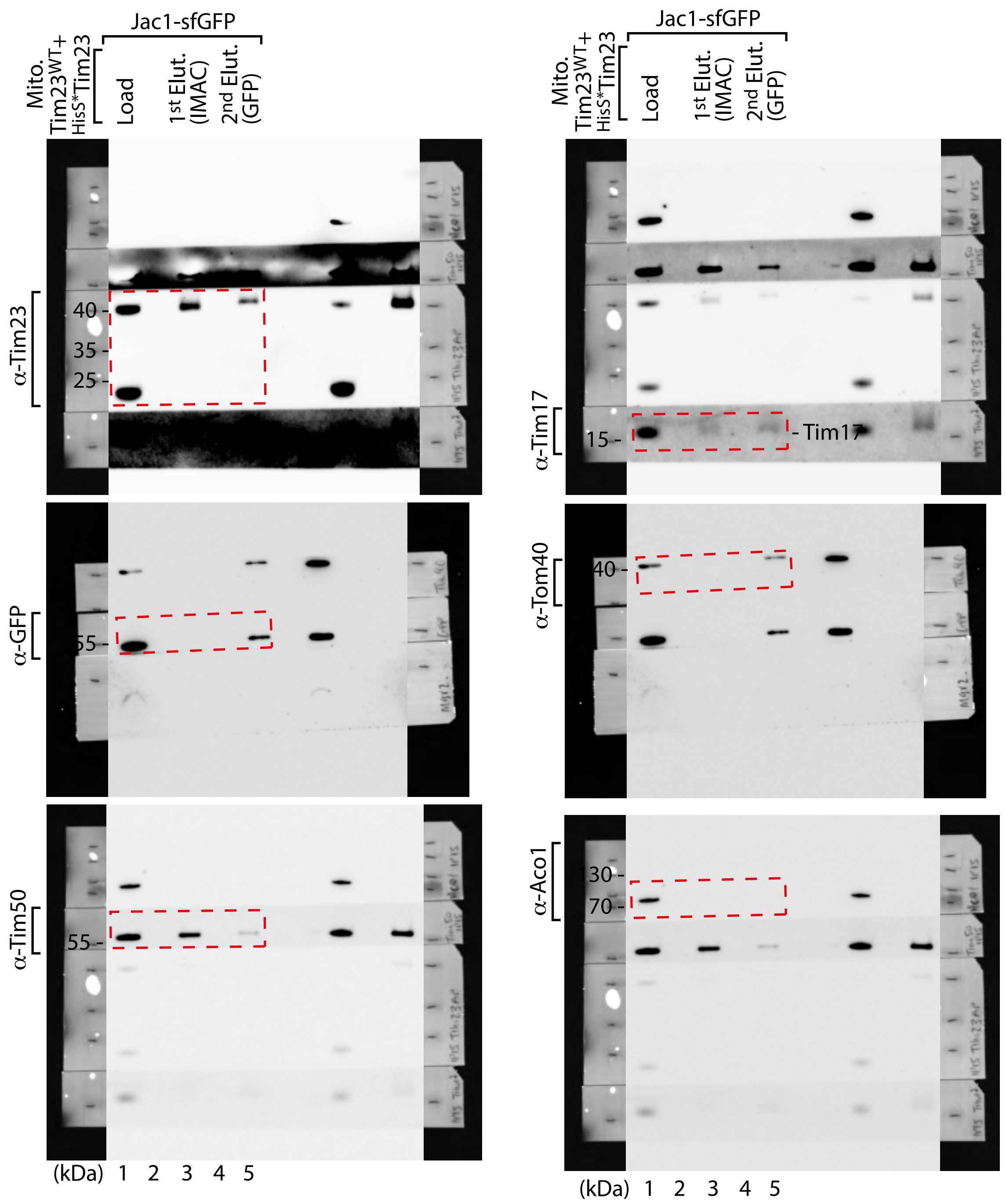

Jac1-sfGFP
Mito. Tim23WT+ HisS*Tim23
Load
1st Elut. (IMAC)
2nd Elut. (GFP)
α-Tim23
40 -
35 -
25 -
α-GFP
55 -
α-Tim50
55 -
(kDa) 1 2 3 4 5
α-Tim17
15 -
- Tim17
α-Tom40
40 -
α-Aco1
30 -
70 -
(kDa) 1 2 3 4 5

Figure 3b

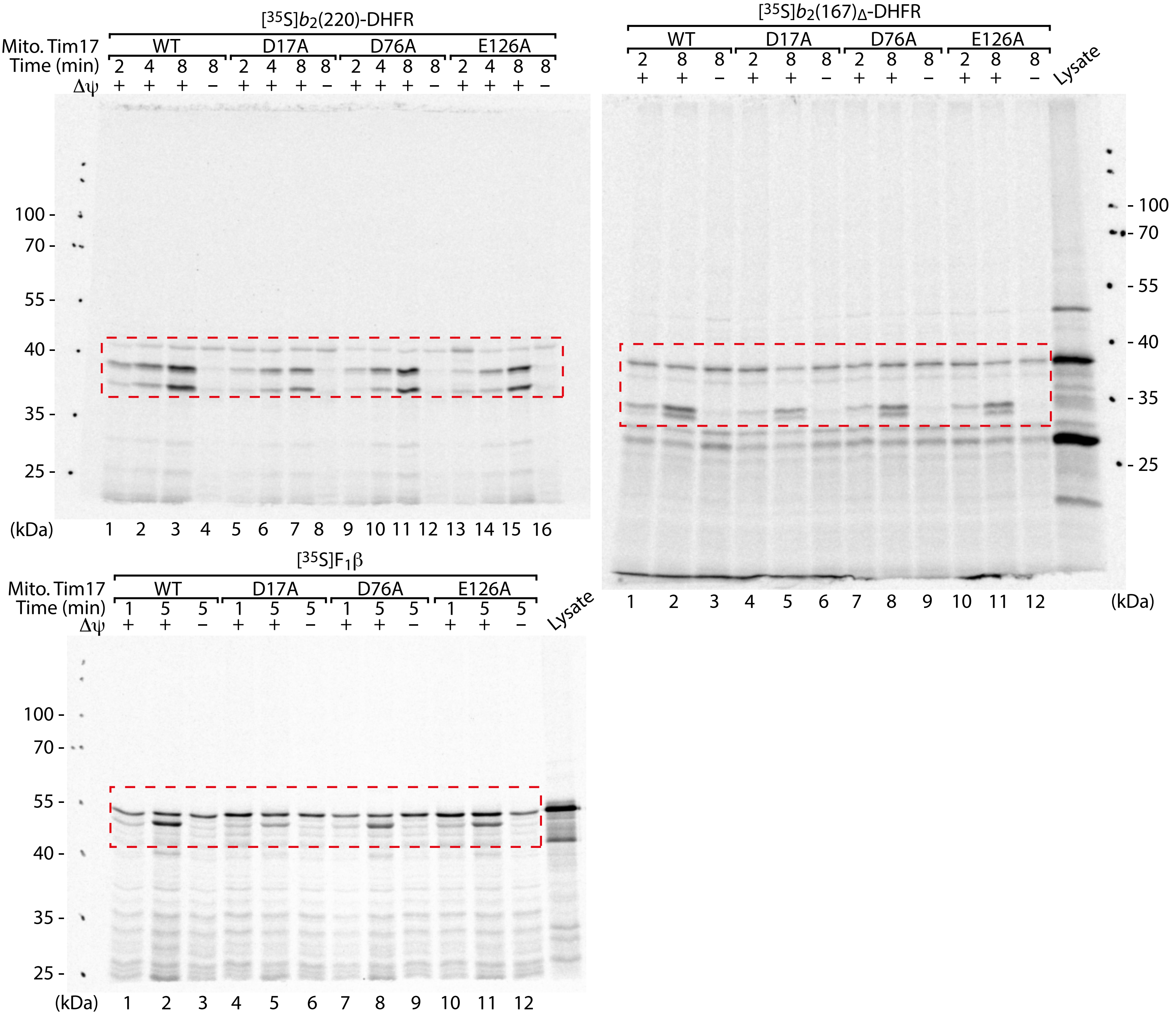

Figure 3c

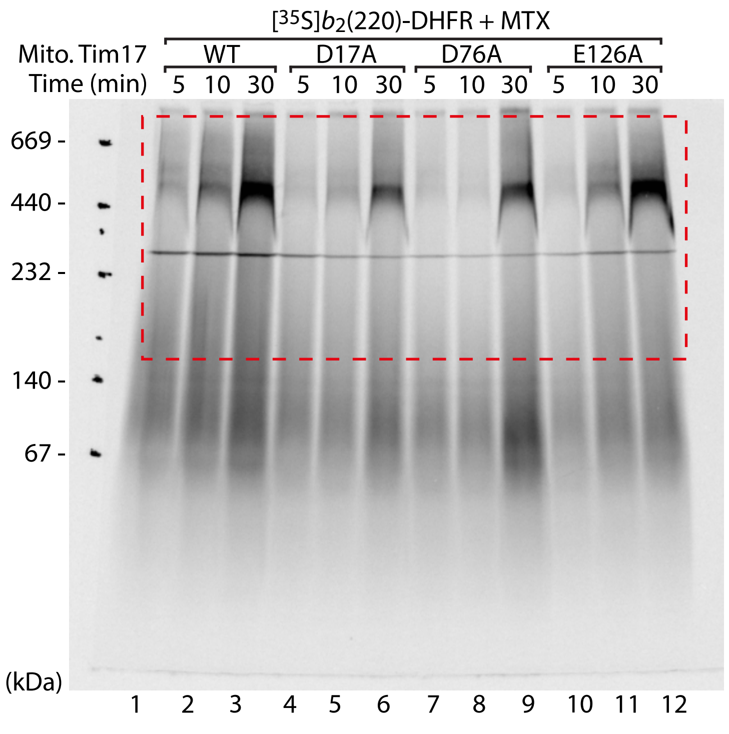

Figure 4b

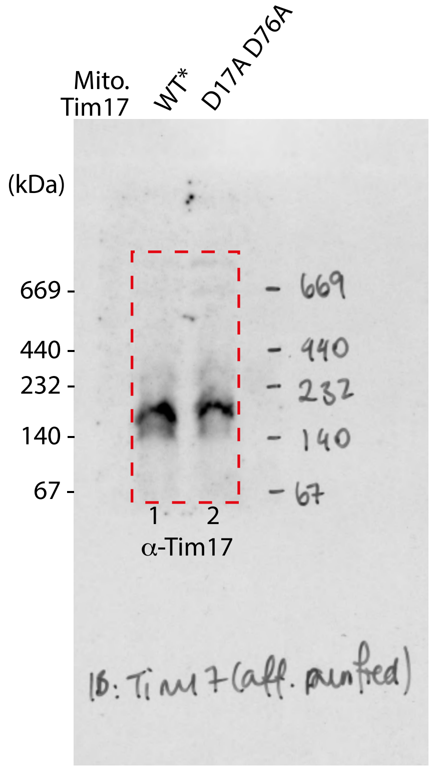

Figure 4c

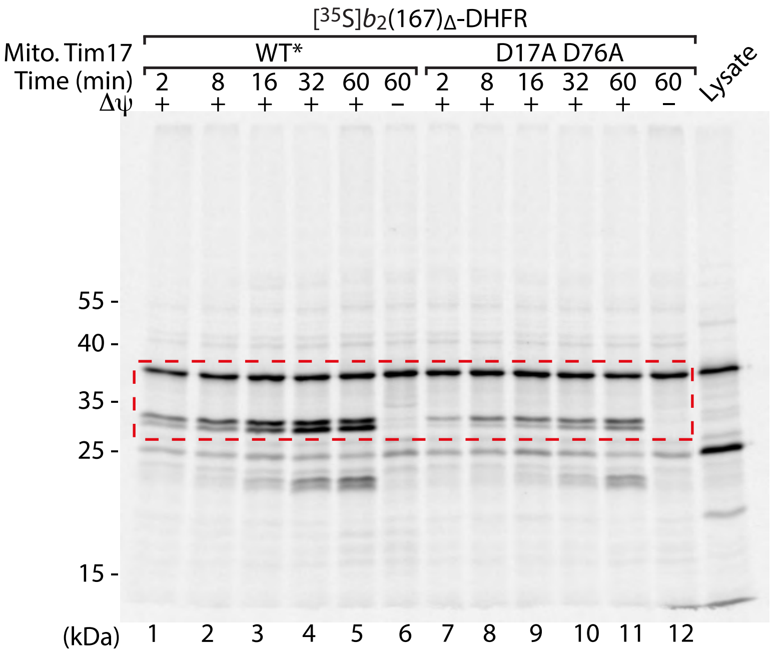

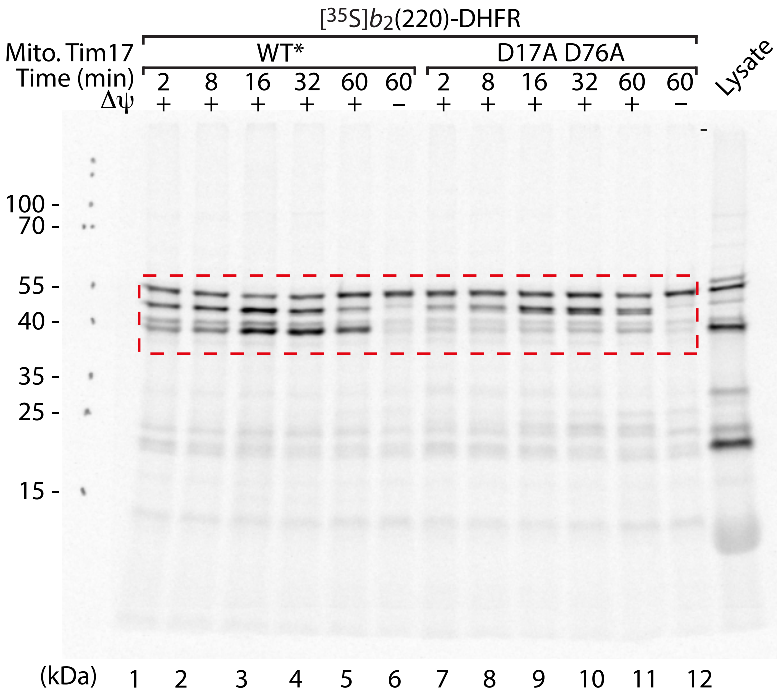

Figure 4d

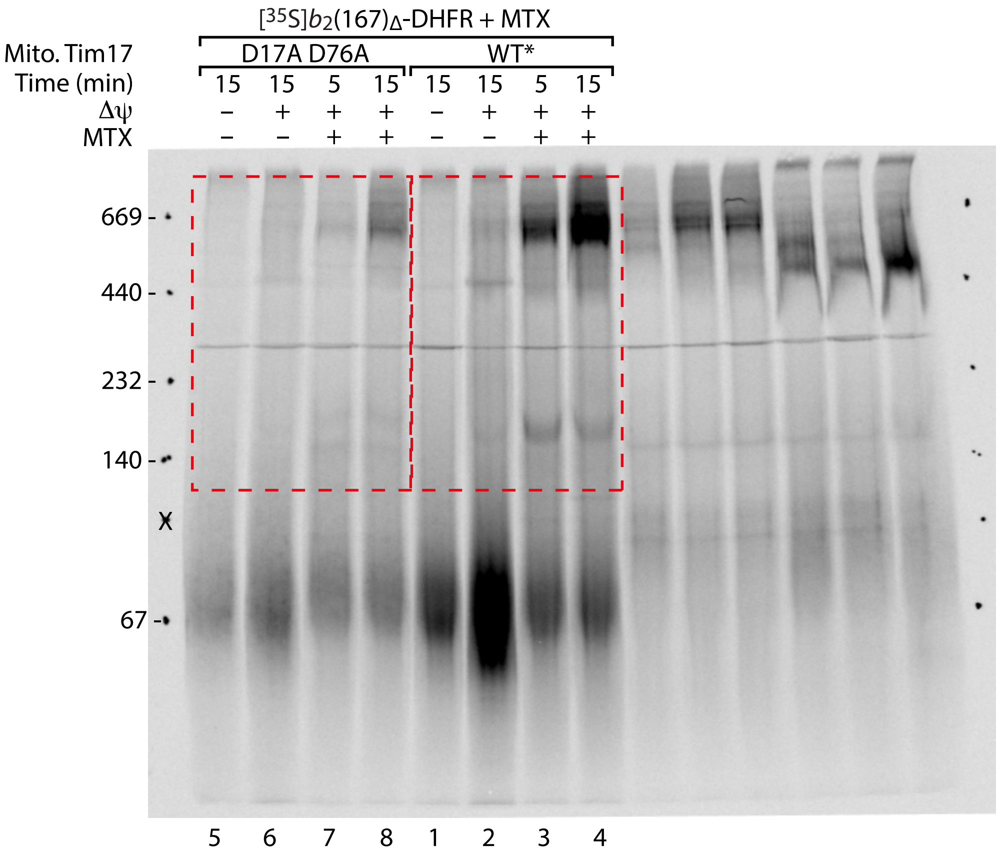

Figure 4e

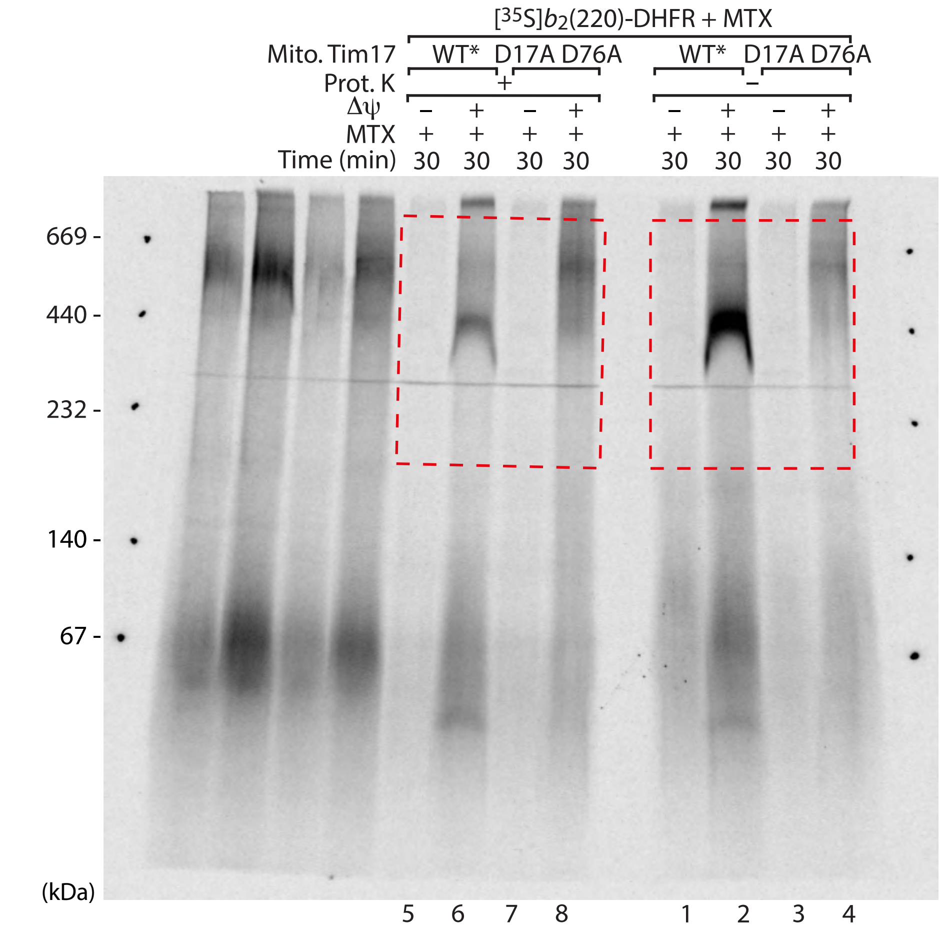

Figure 5b

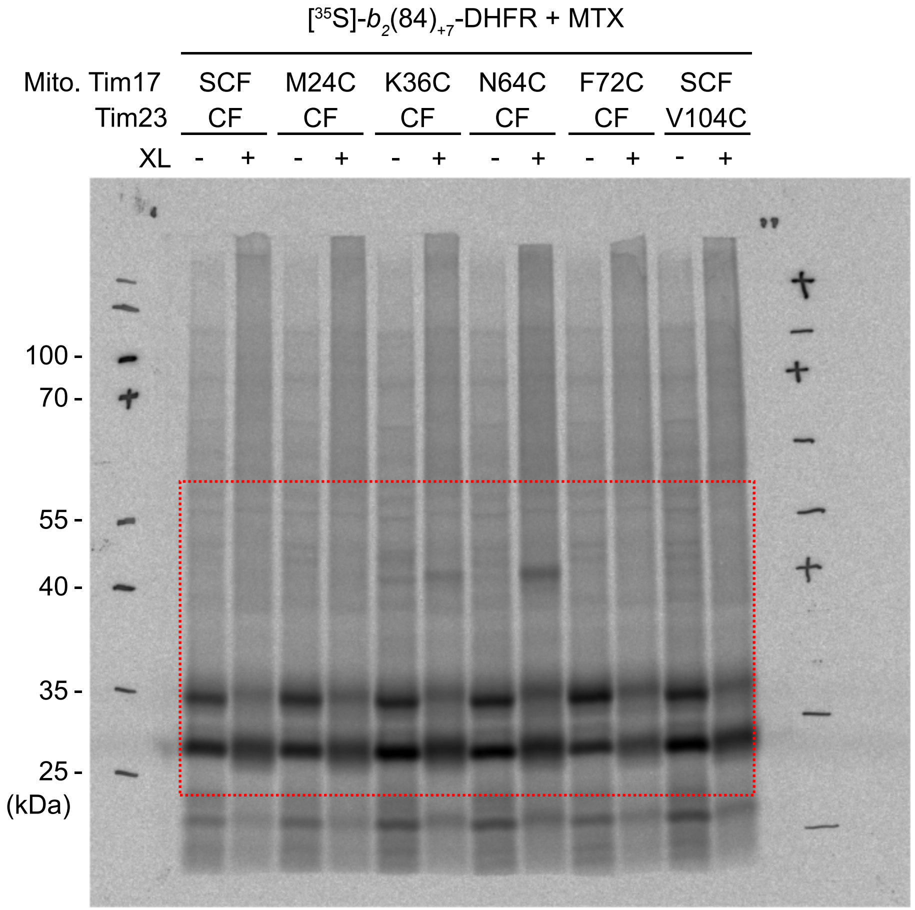

Figure 5c

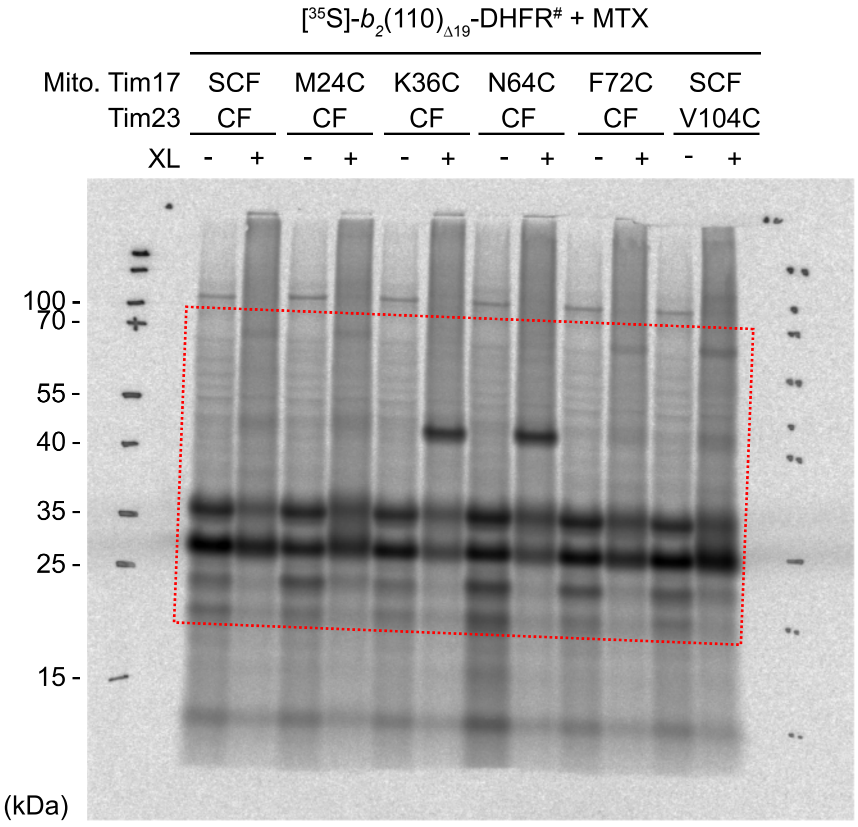

Figure 5d

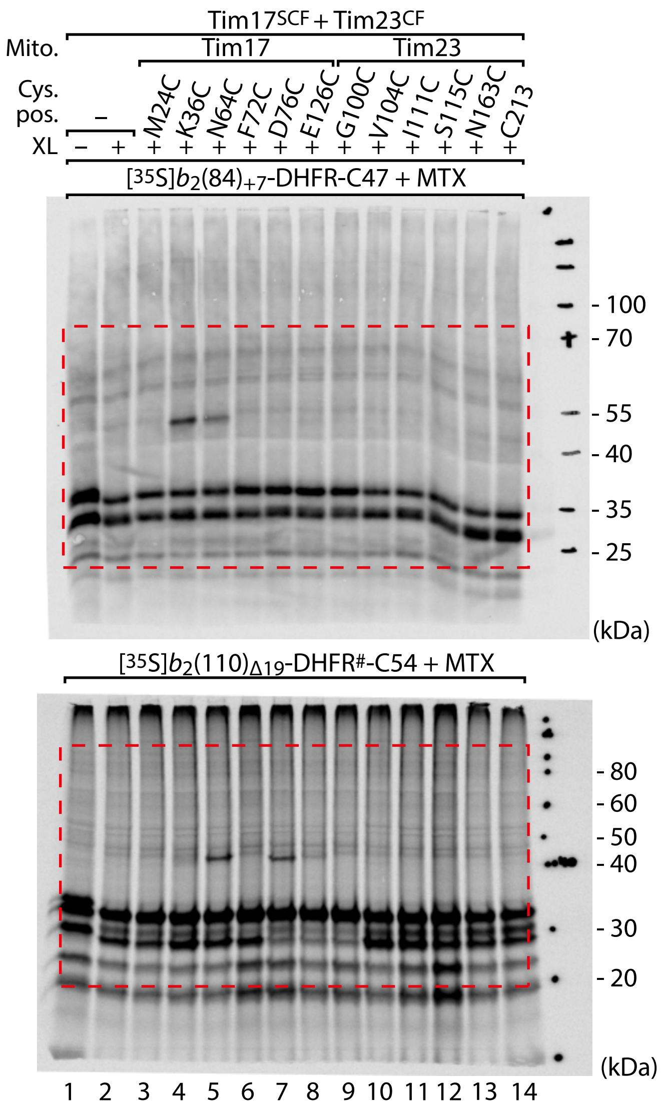

Tim17SCF + Tim23CF
Mito.
Tim17
Tim23
Cys. pos.
–
M24C
K36C
N64C
F72C
D76C
E126C
G100C
V104C
I111C
S115C
N163C
C213
XL – + + + + + + + + + + + + +
[35S]b2(84)+7-DHFR-C47 + MTX
- 100
- 70
- 55
- 40
- 35
- 25
(kDa)
[35S]b2(110)Δ19-DHFR#-C54 + MTX
- 80
- 60
- 50
- 40
- 30
- 20
(kDa)
1 2 3 4 5 6 7 8 9 10 11 12 13 14

Extended Data Figure 1a

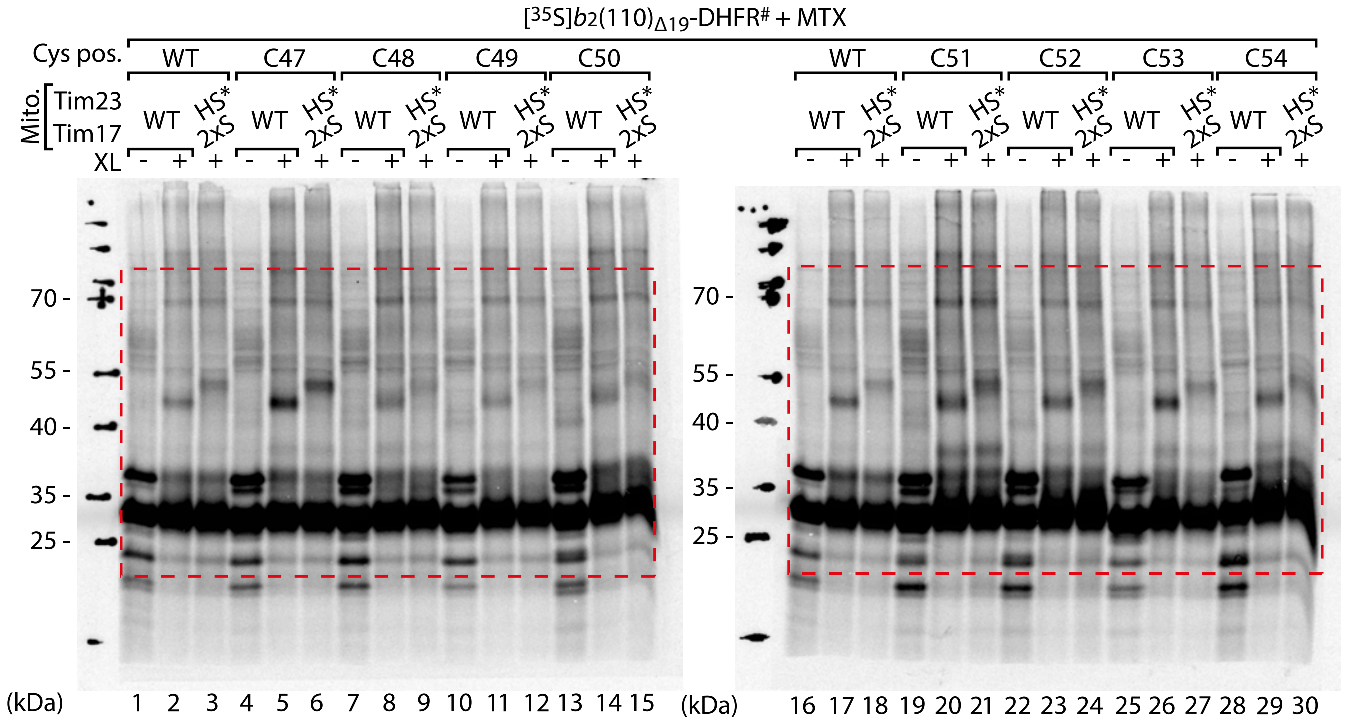

Extended Data Figure 1b

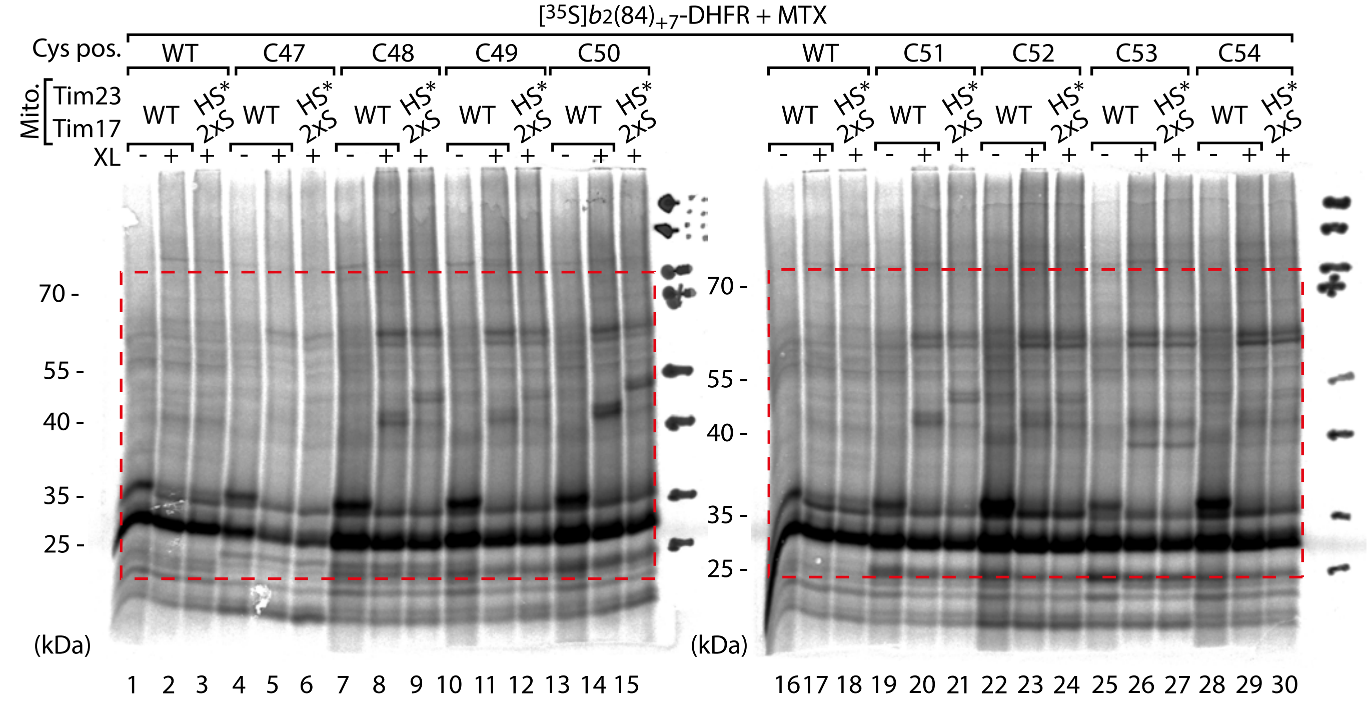

Extended Data Figure 1c

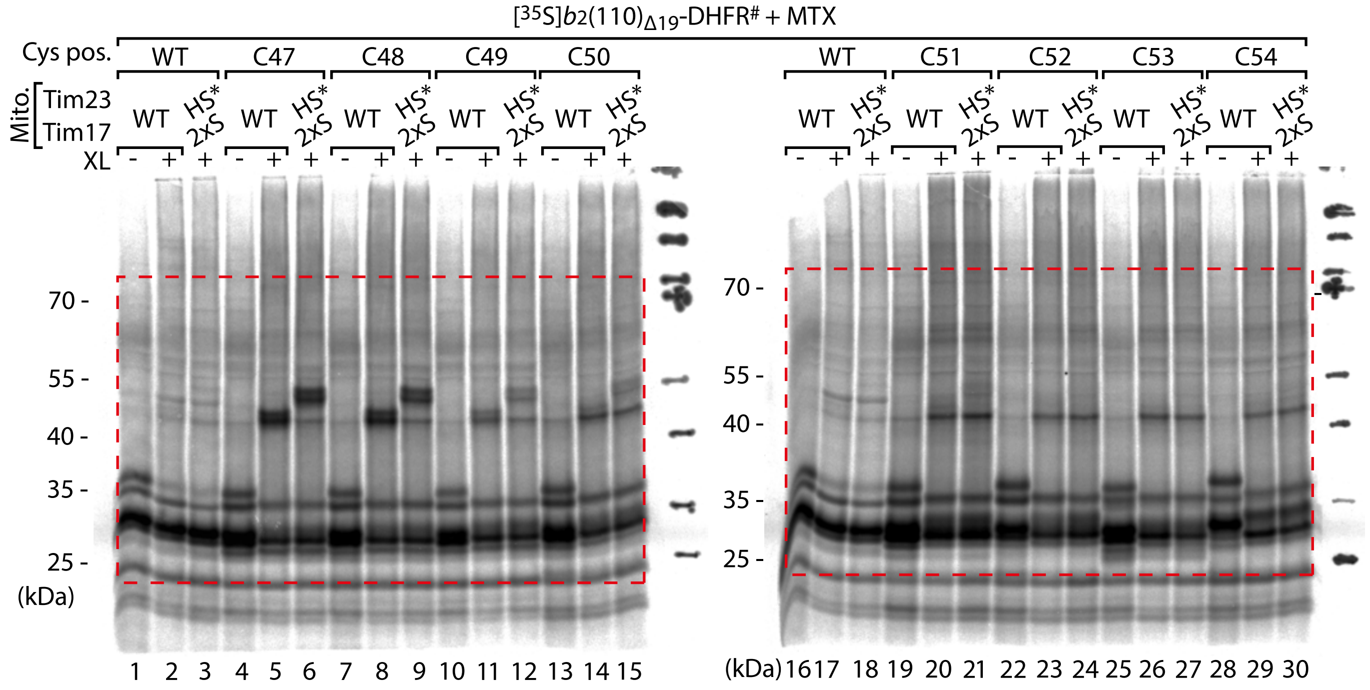

Extended Data Figure 1d

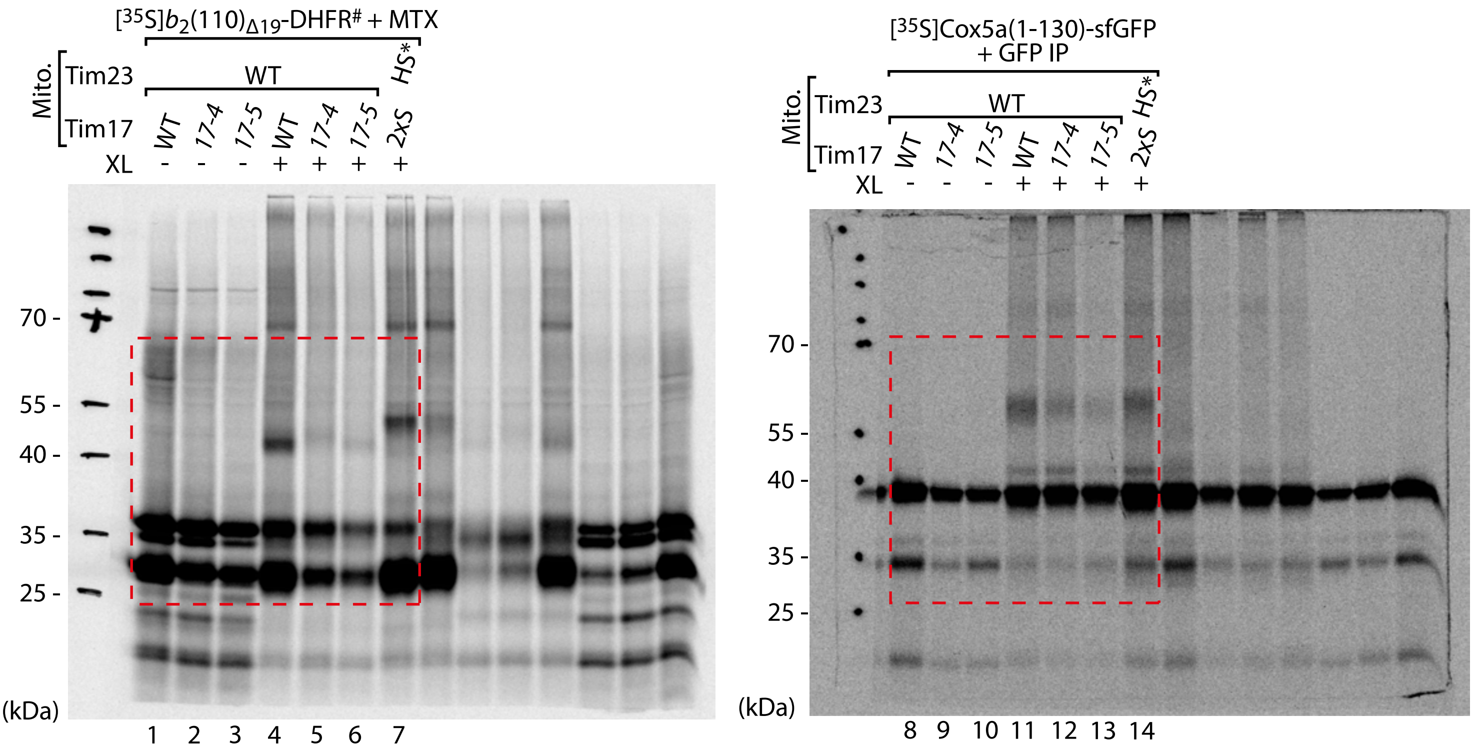

Extended Data figure 2c

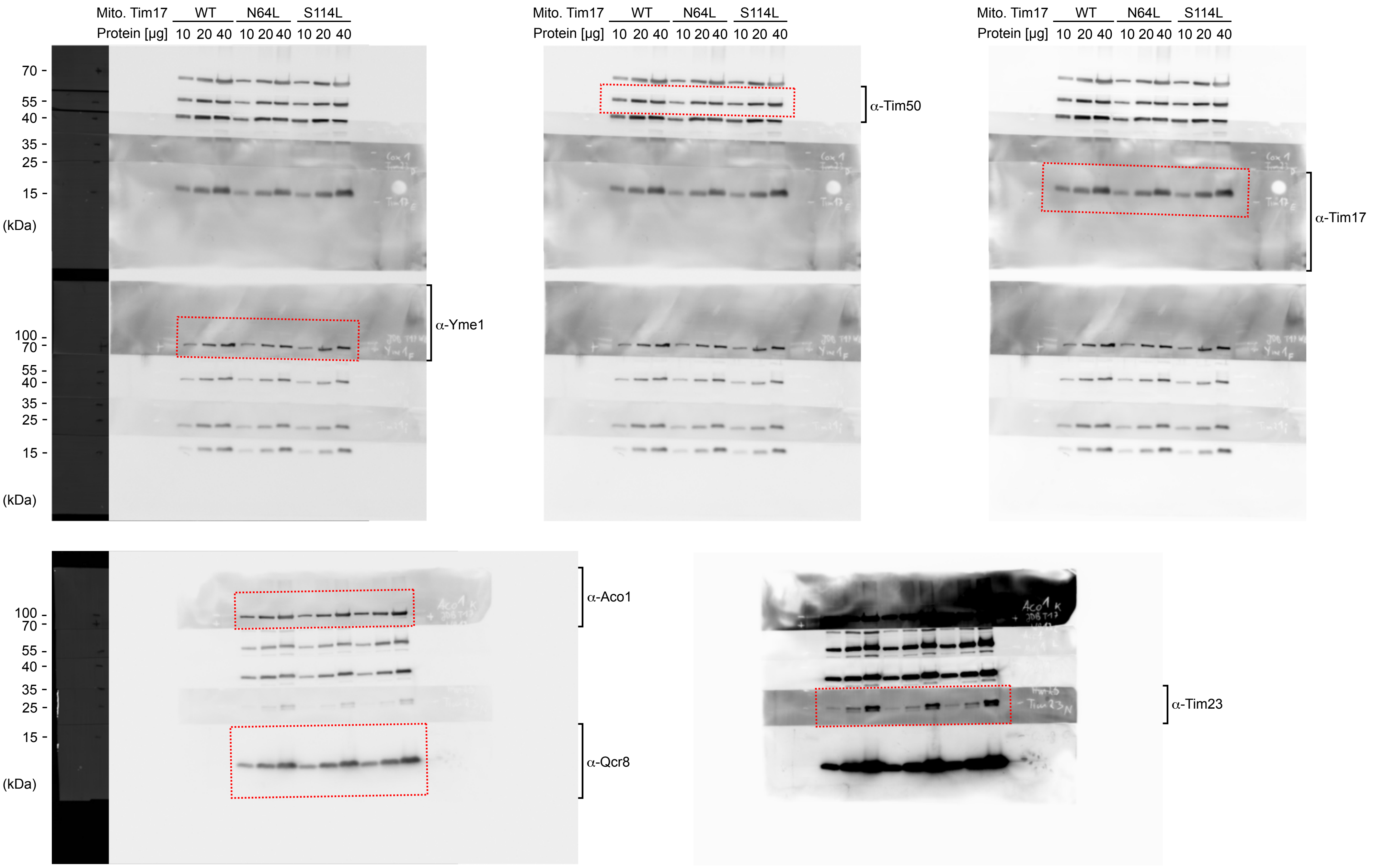

Extended Data figure 2e

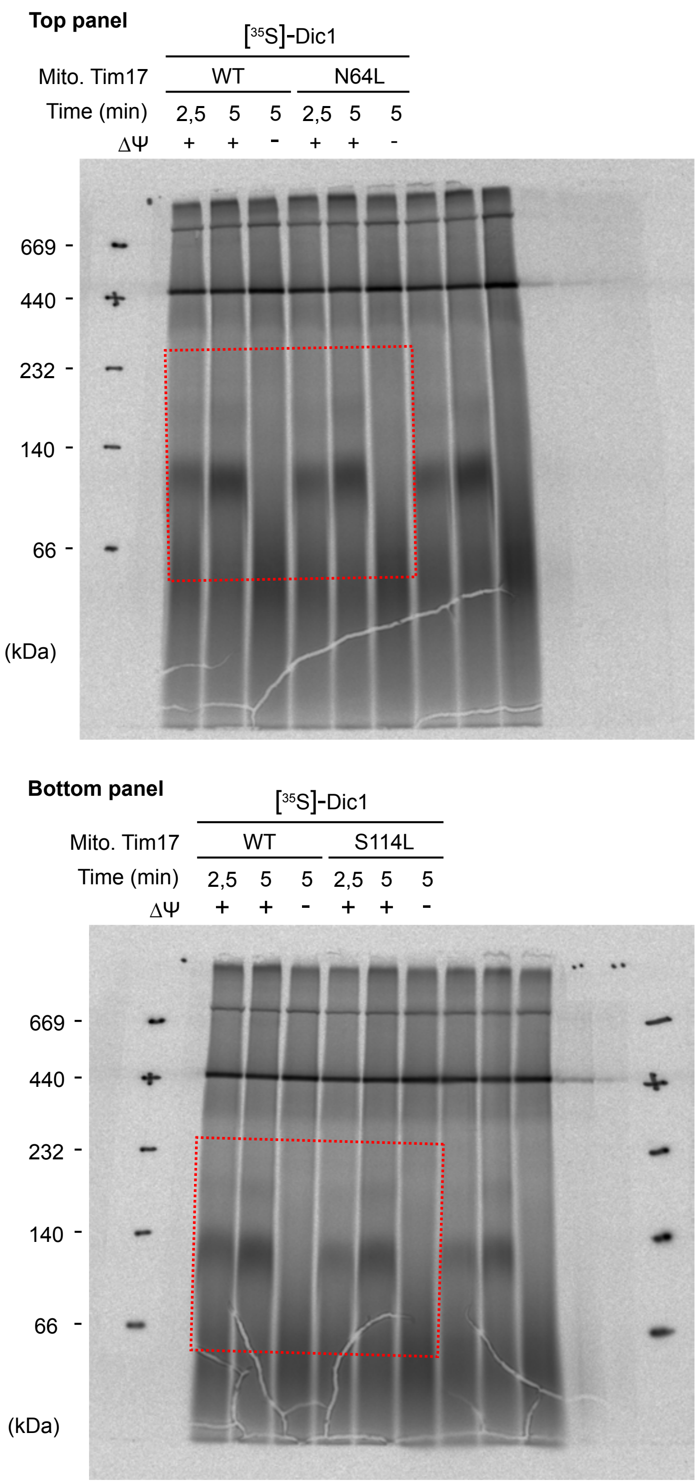

Extended Data figure 2f

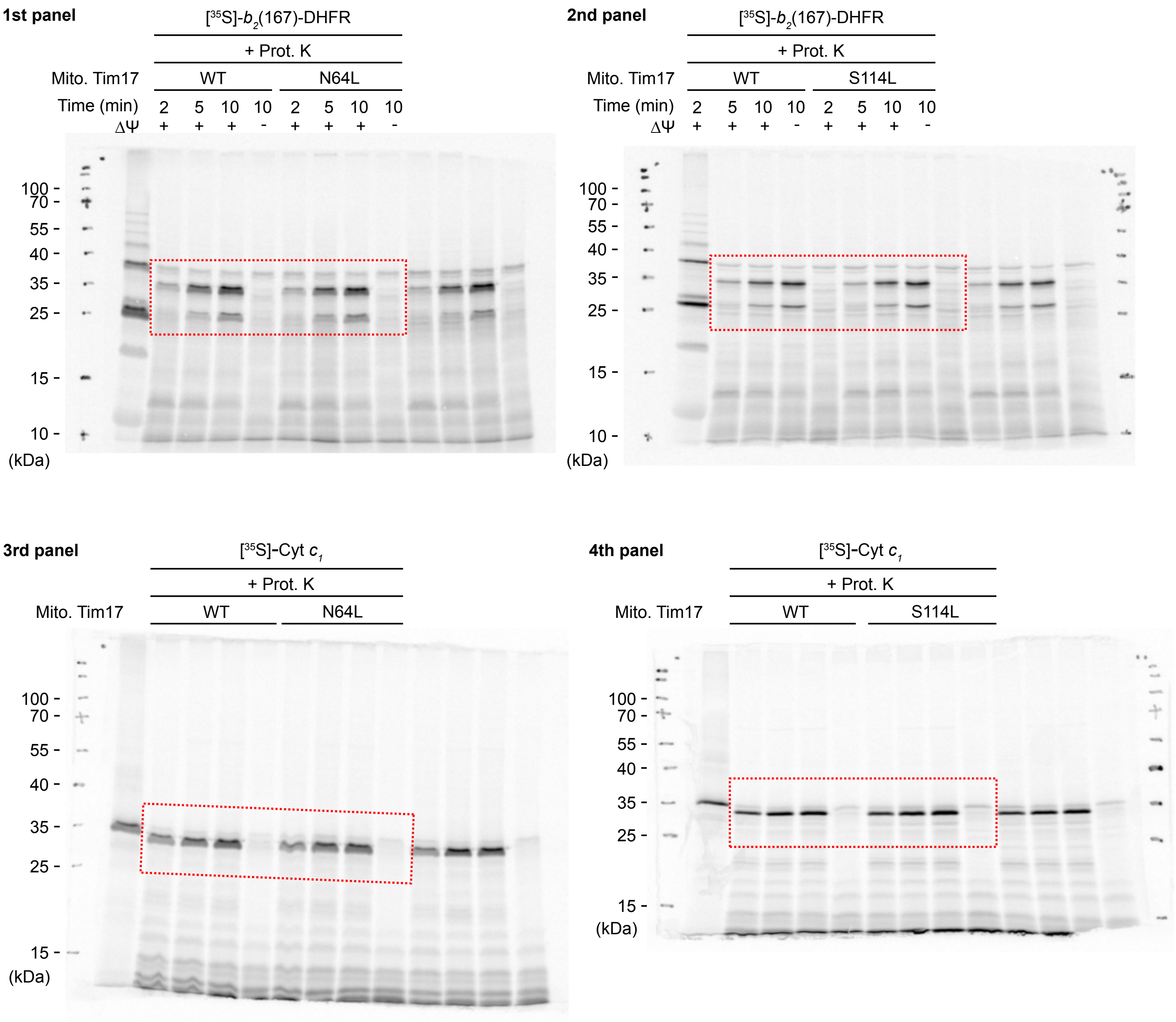

Extended Data Figure 3d

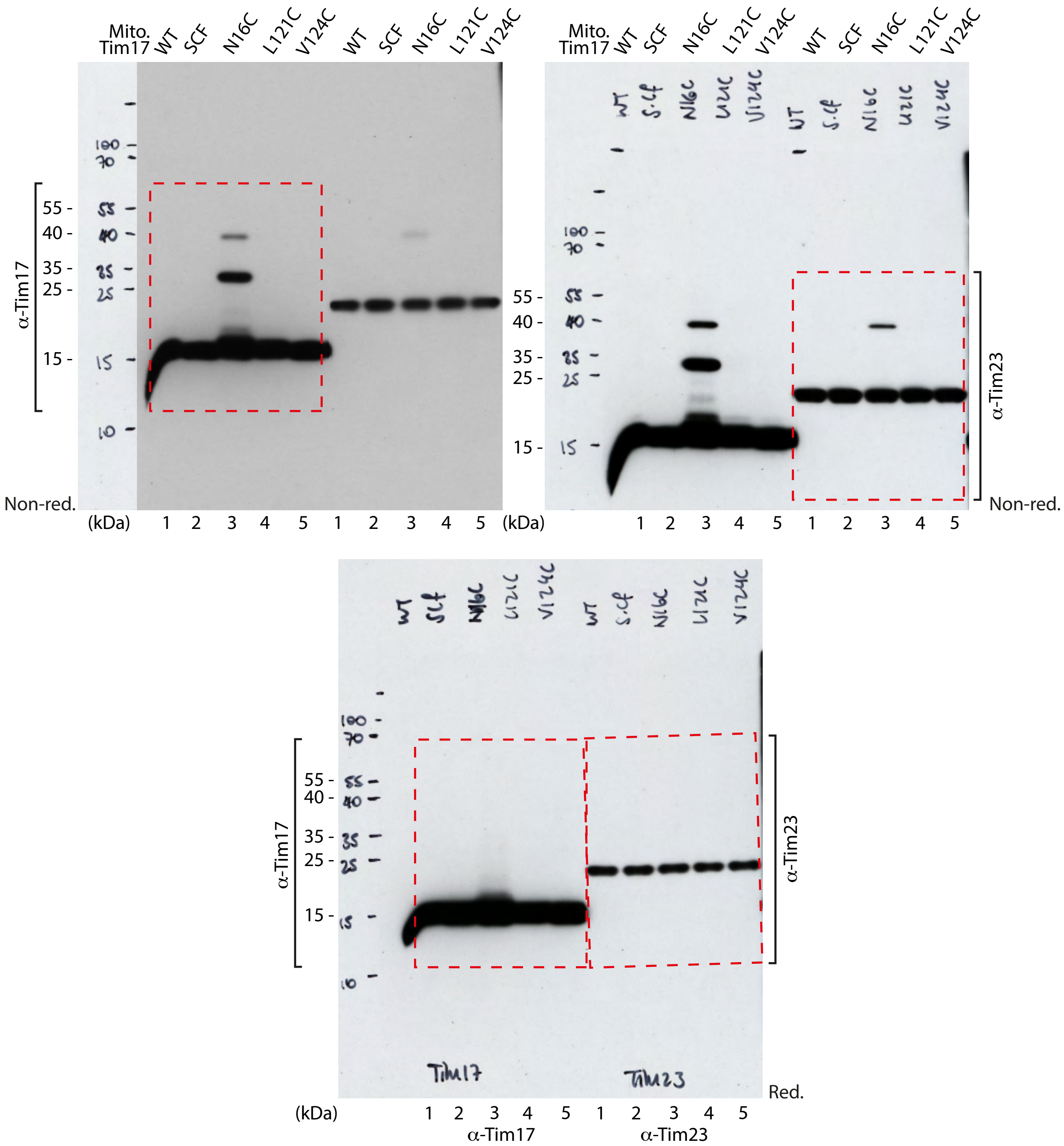

Extended Data Figure 3f

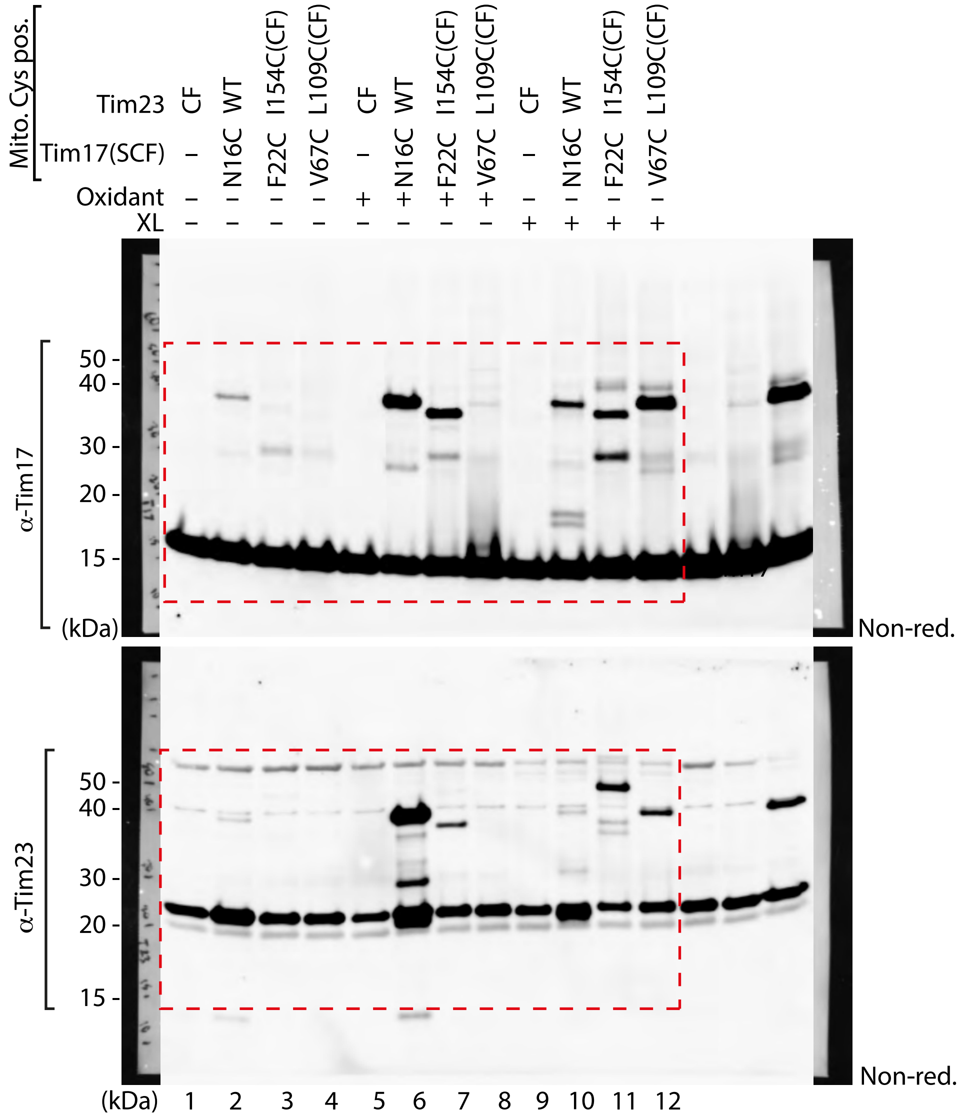

Extended Data Figure 6a

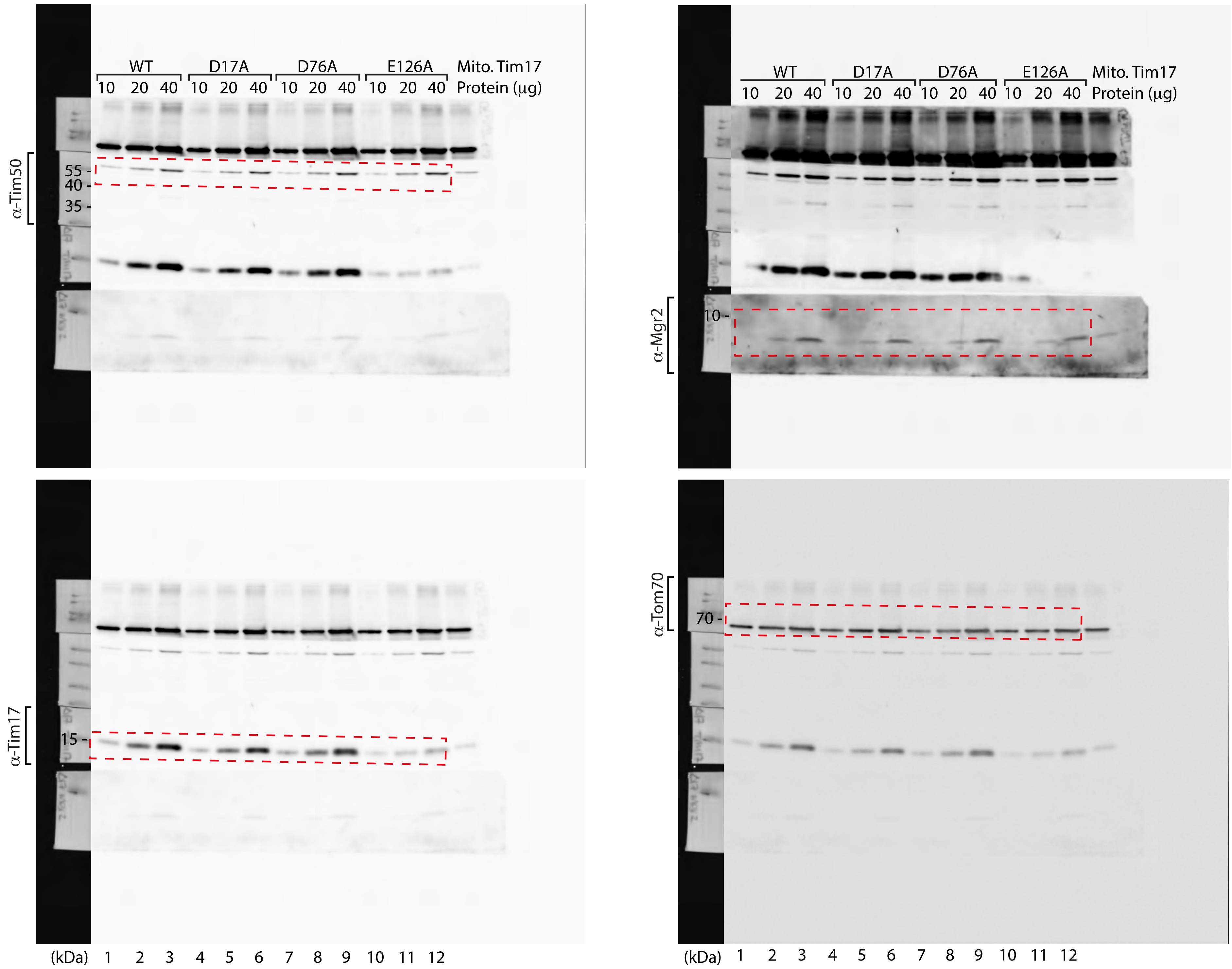

Extended Data Figure 6a

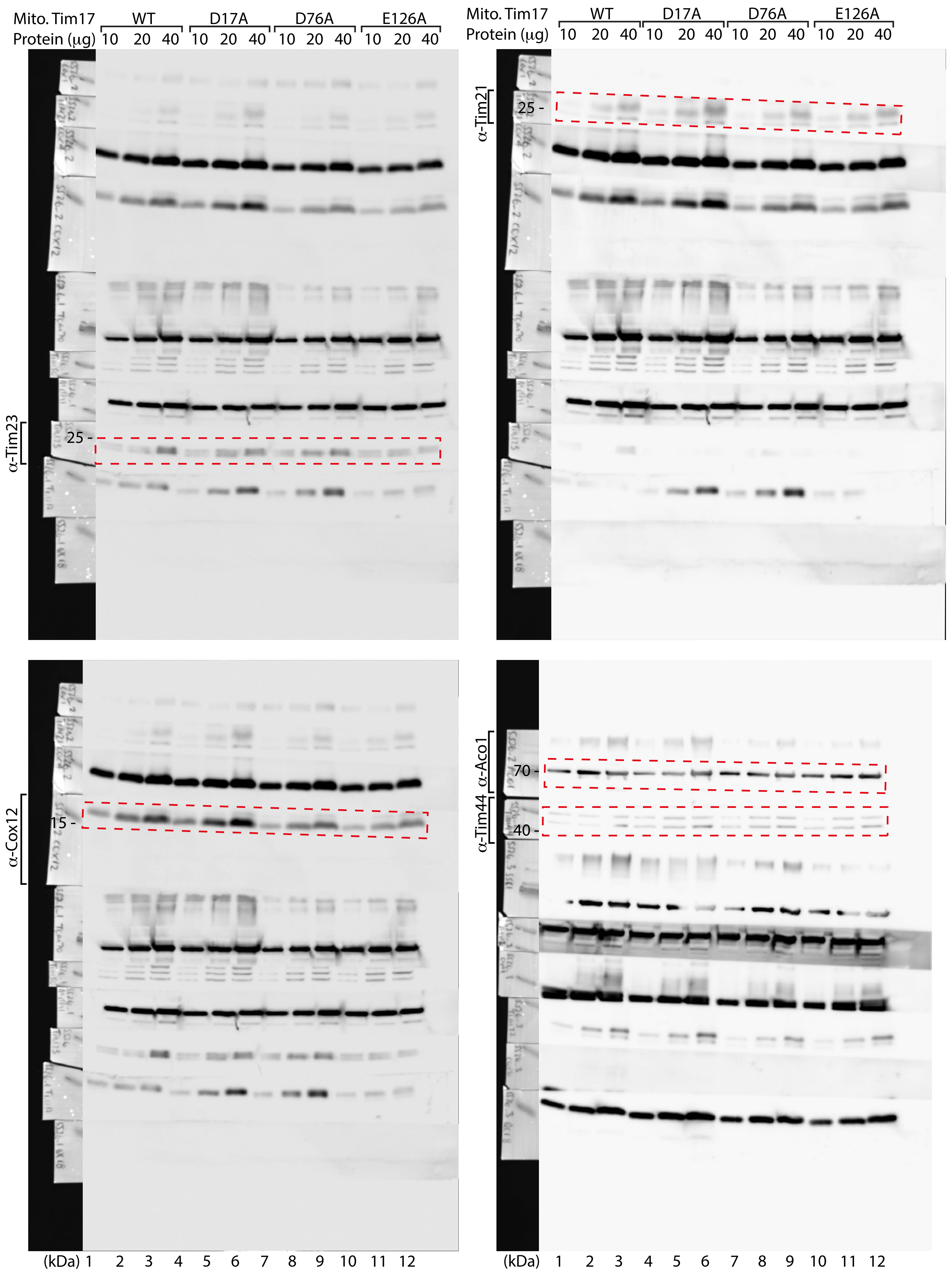

Extended Data Figure 6a

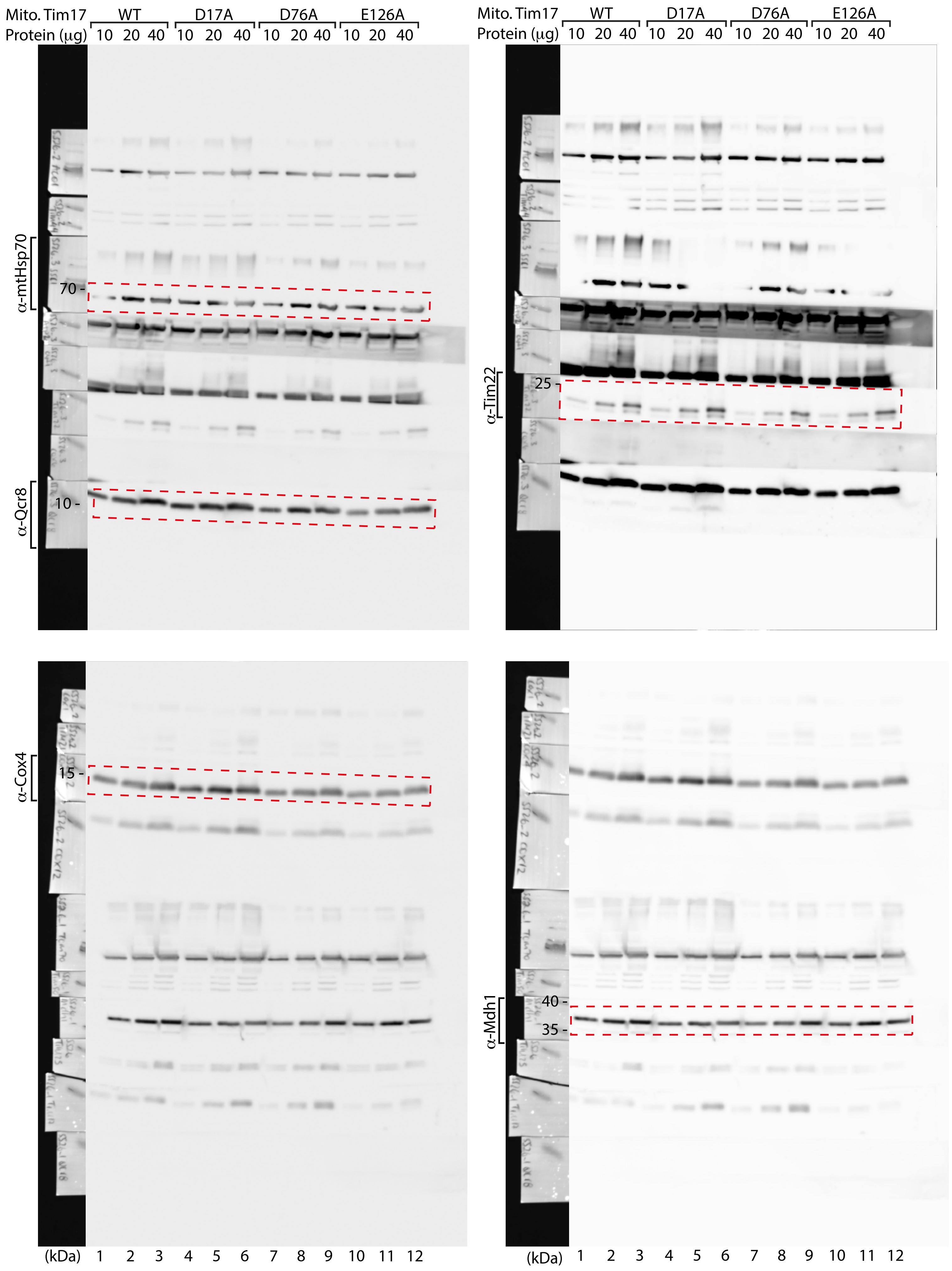

Extended Data Figure 6b

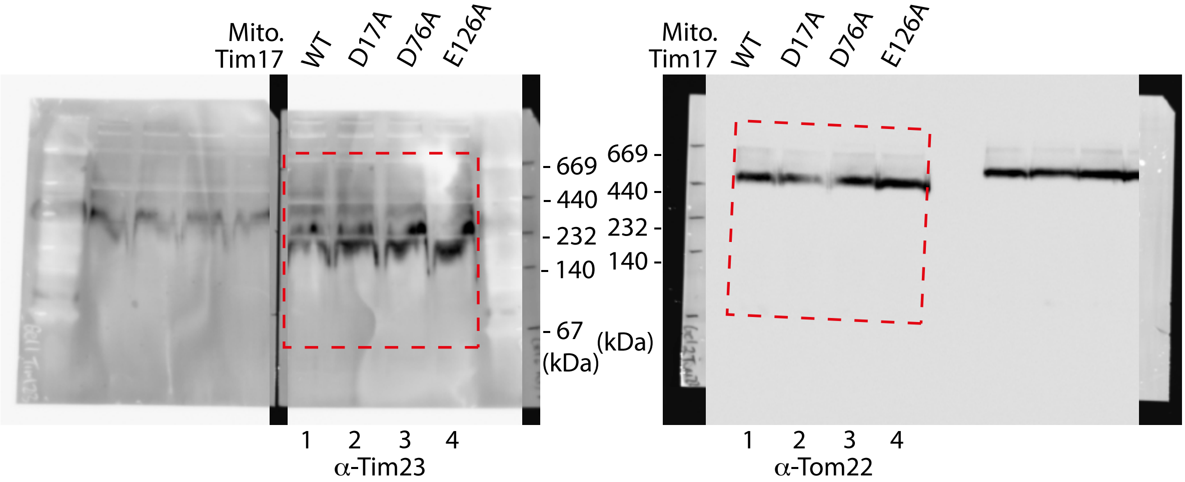

Extended Data Figure 6d

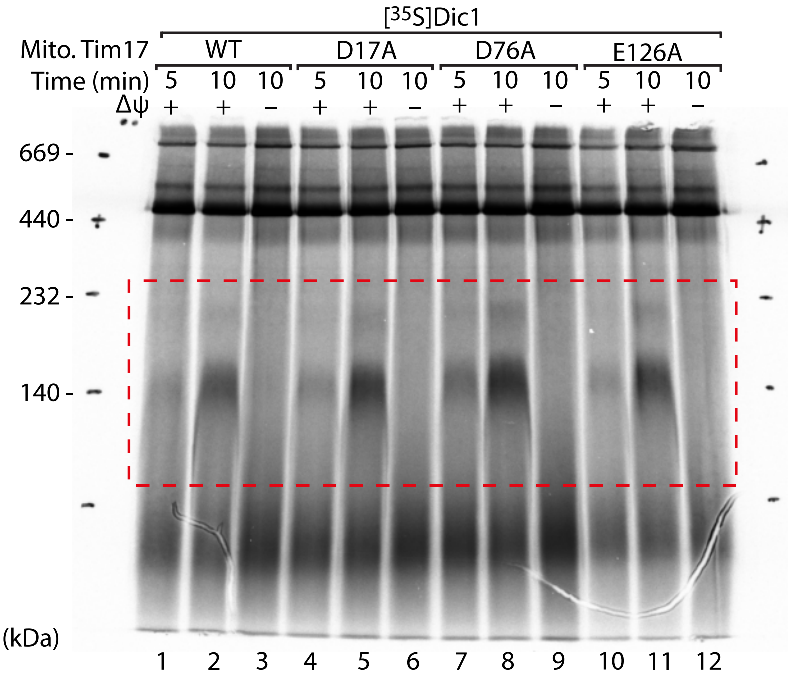

Extended data Figure 6f

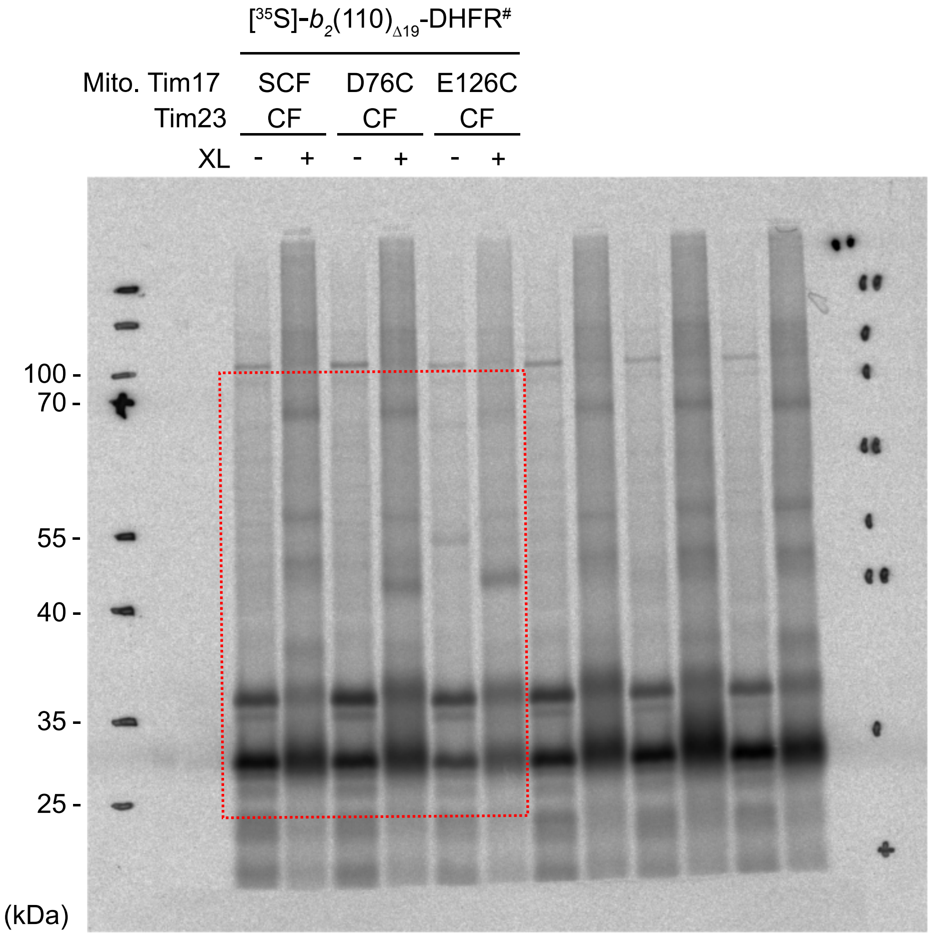

Extended Data Figure 7b

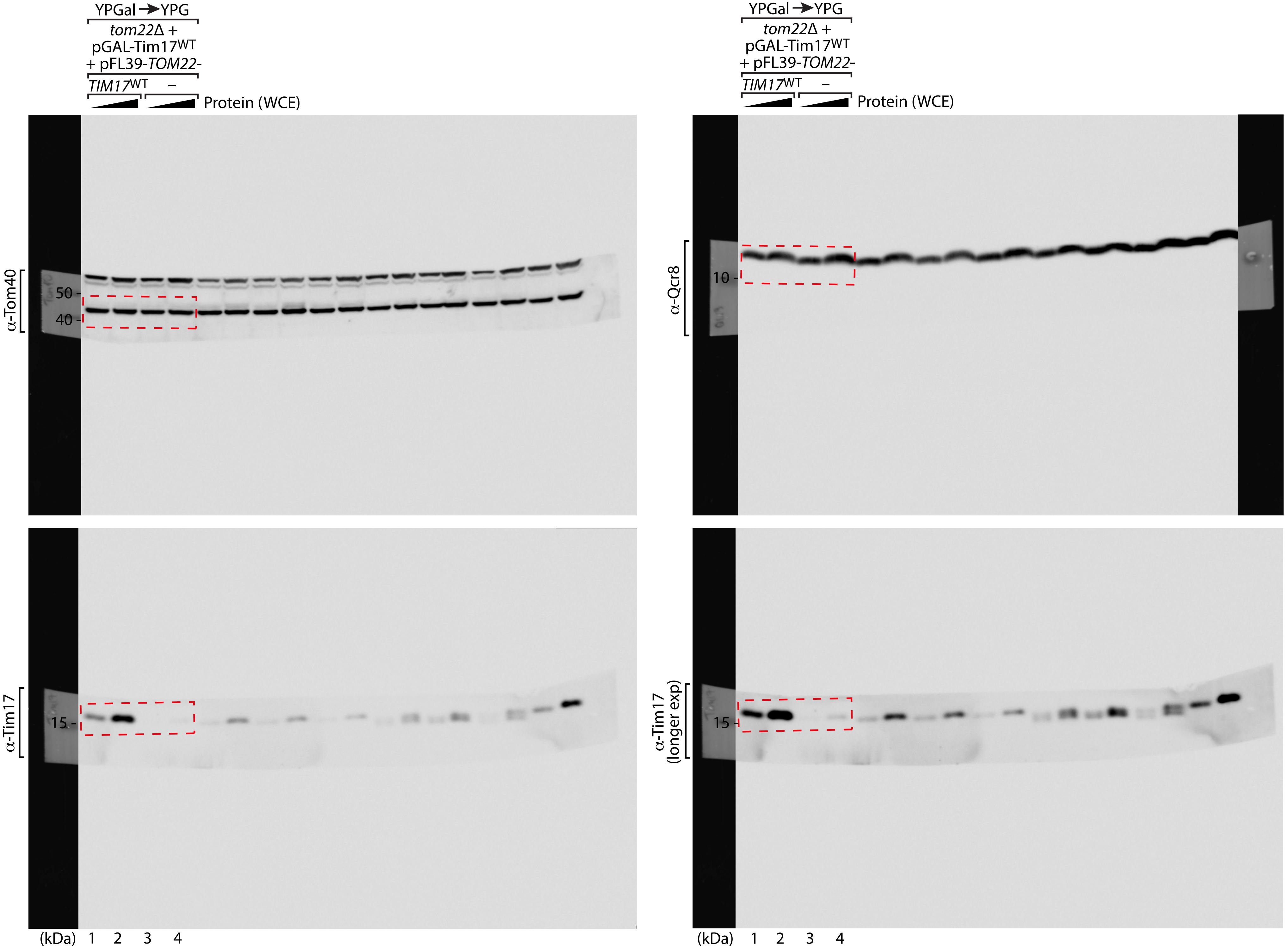

Extended Data Figure 7d

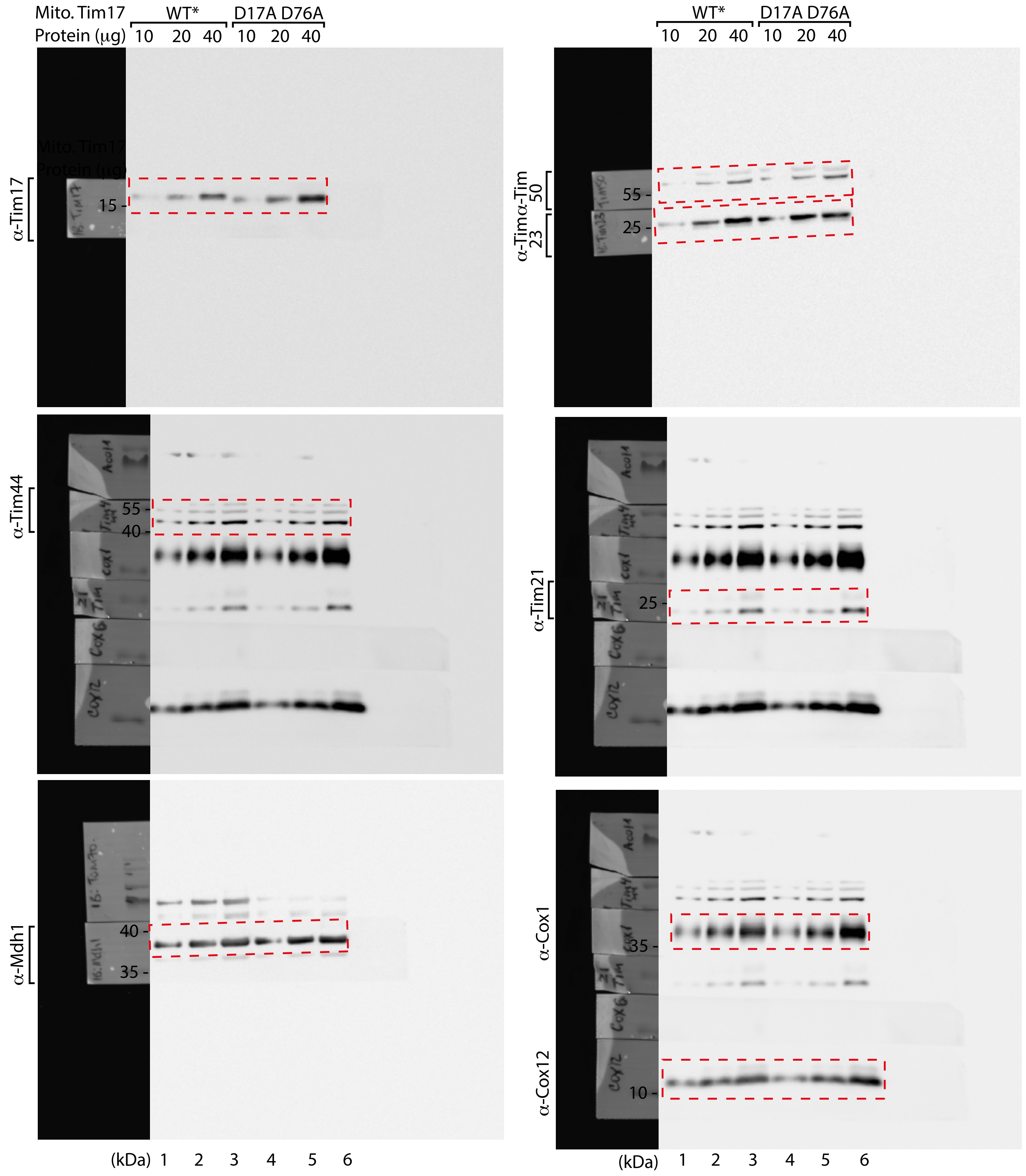

Extended Data Figure 7e

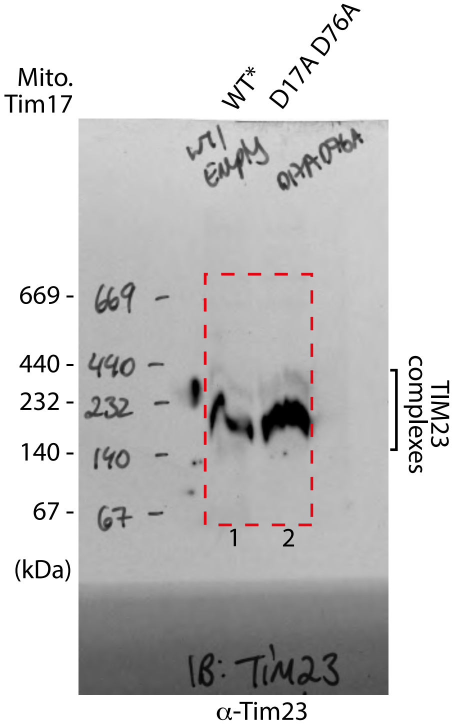

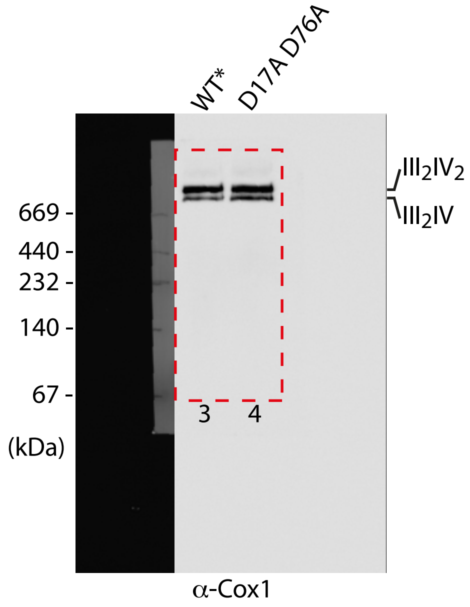

Extended Data Figure 7f

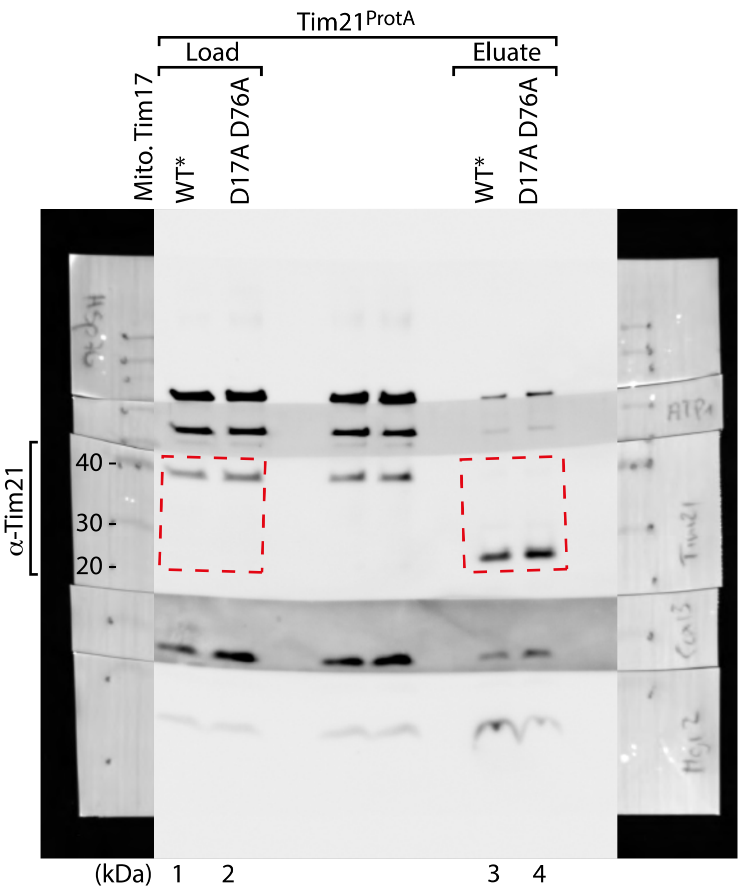

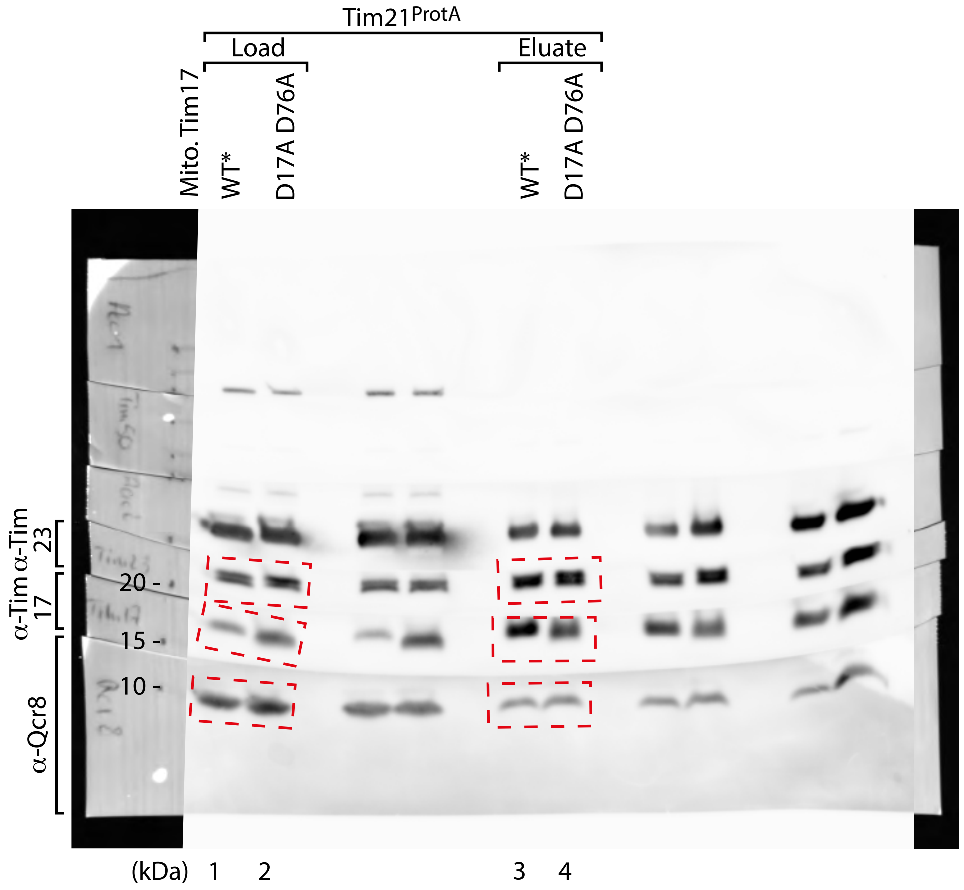

Extended Data Figure 7f cont.

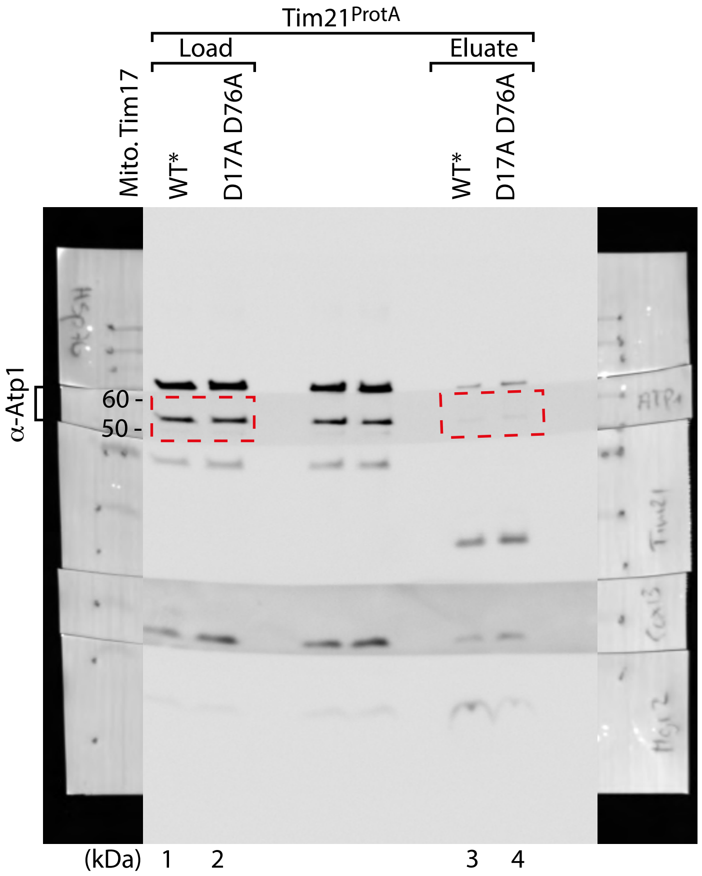

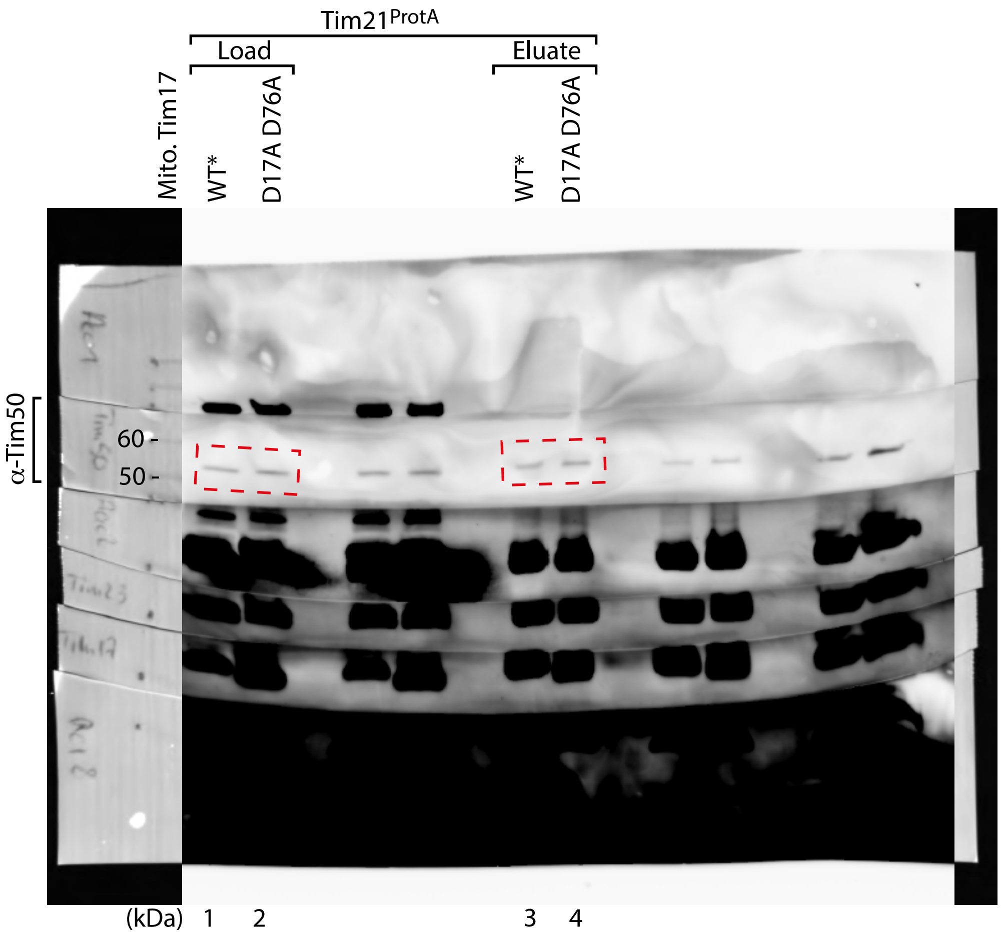

Extended Data Figure 7f cont.

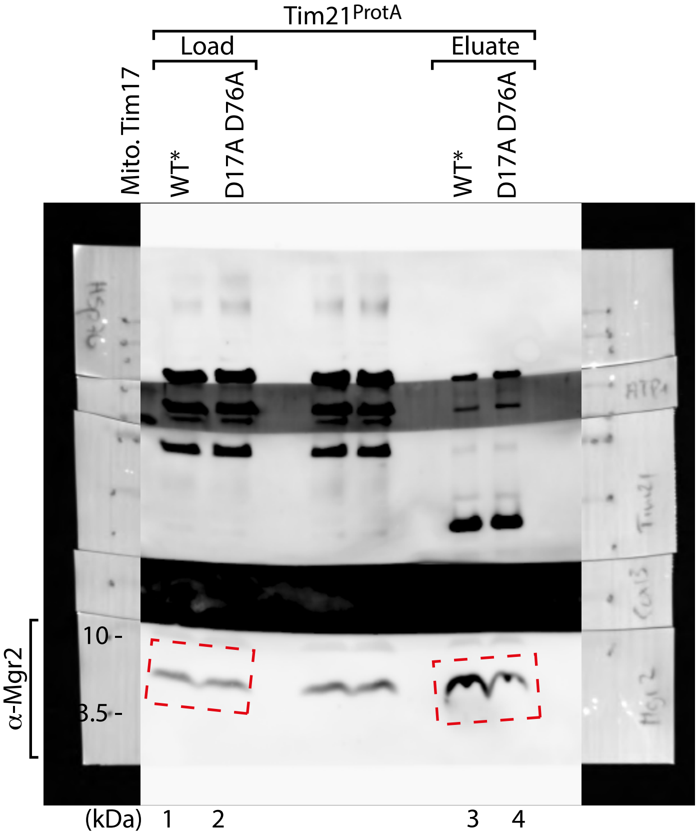

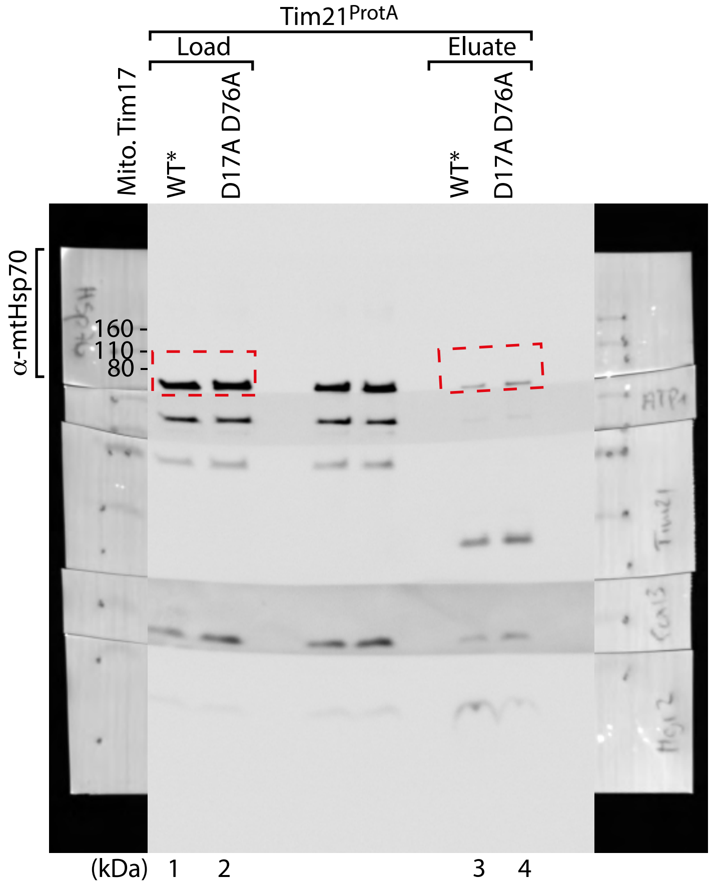

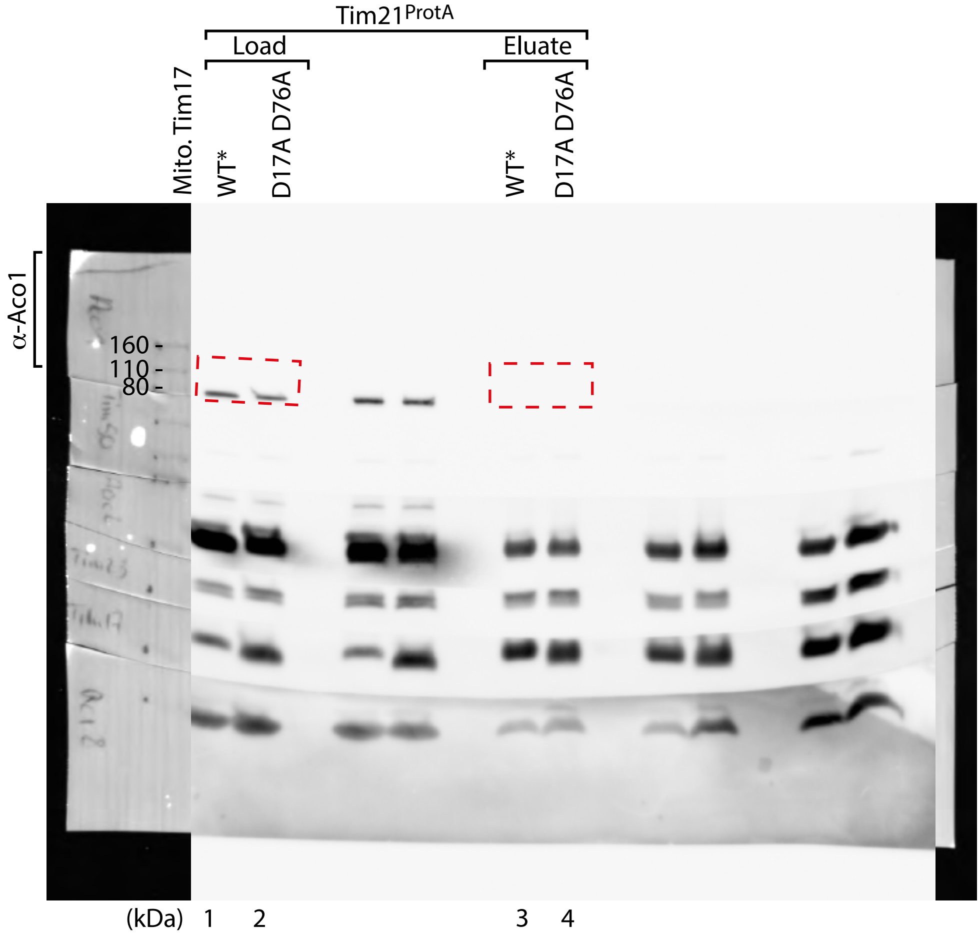

Extended Data Figure 7h

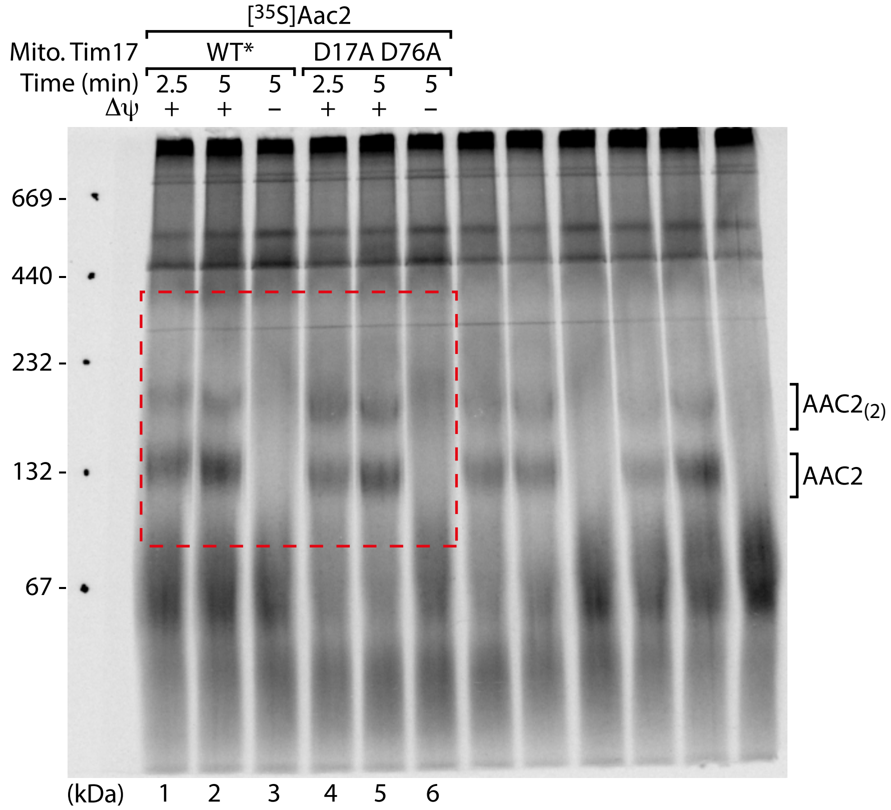

Extended Data Figure 7i

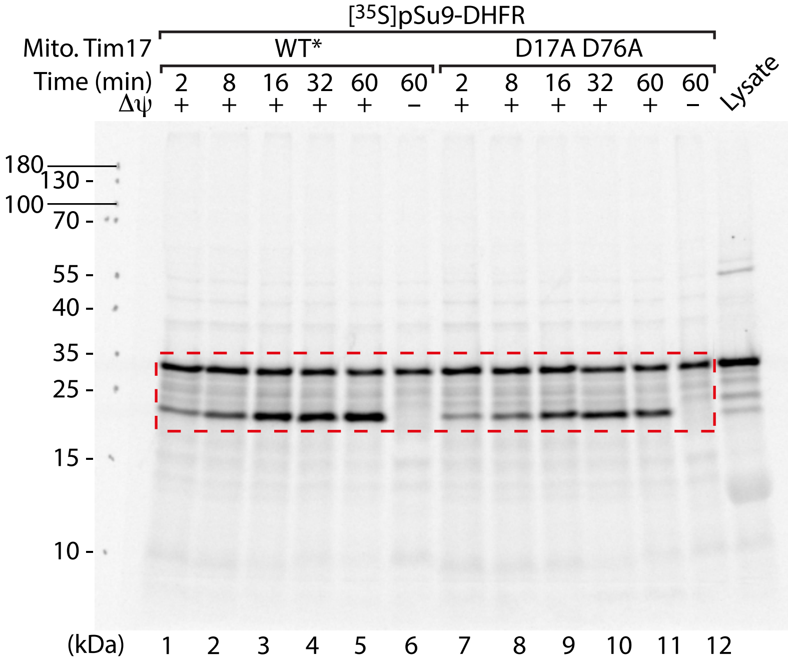

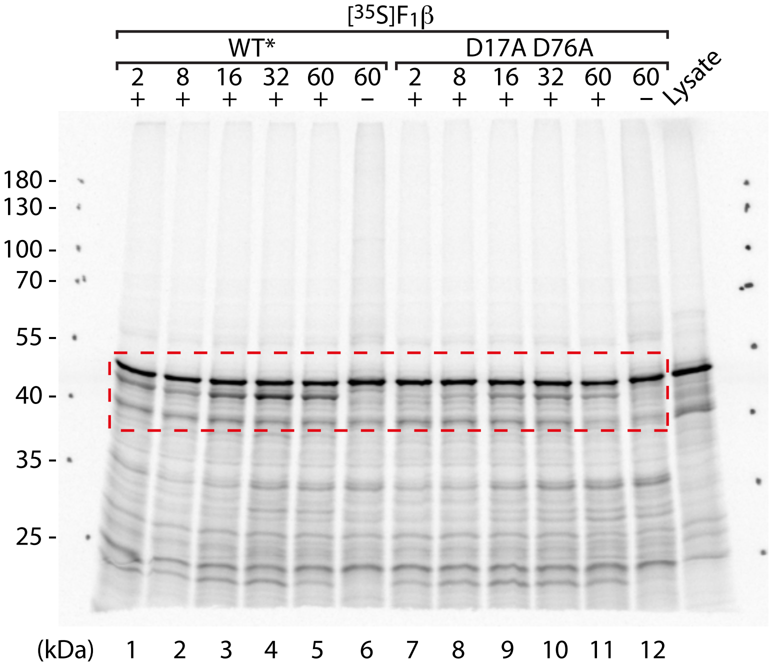

Extended Data Figure 8b

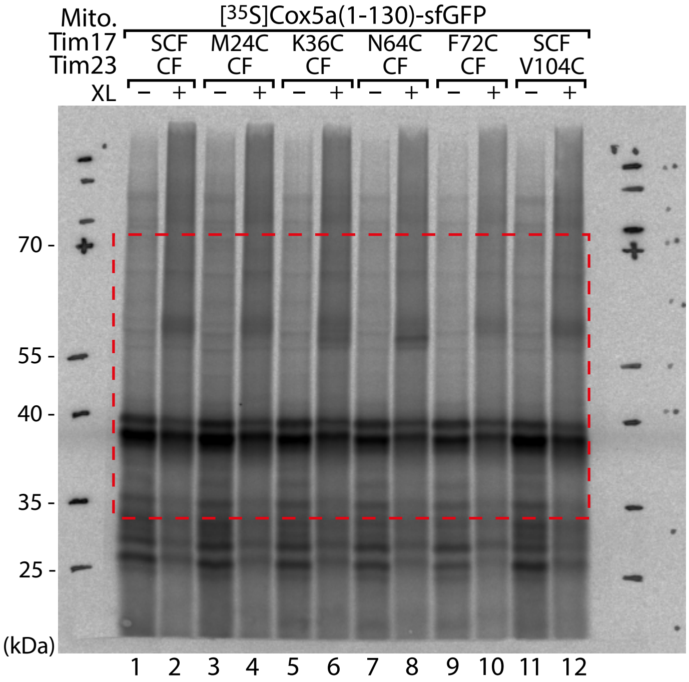

Extended data Figure 8c

left panel

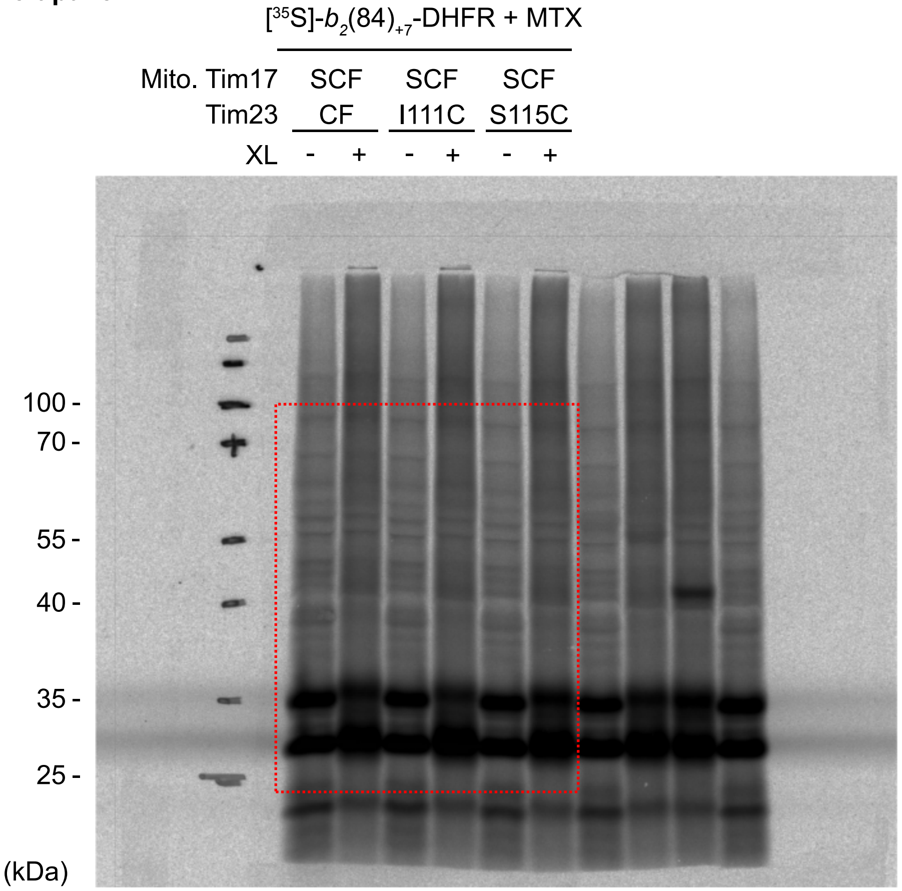

right panel

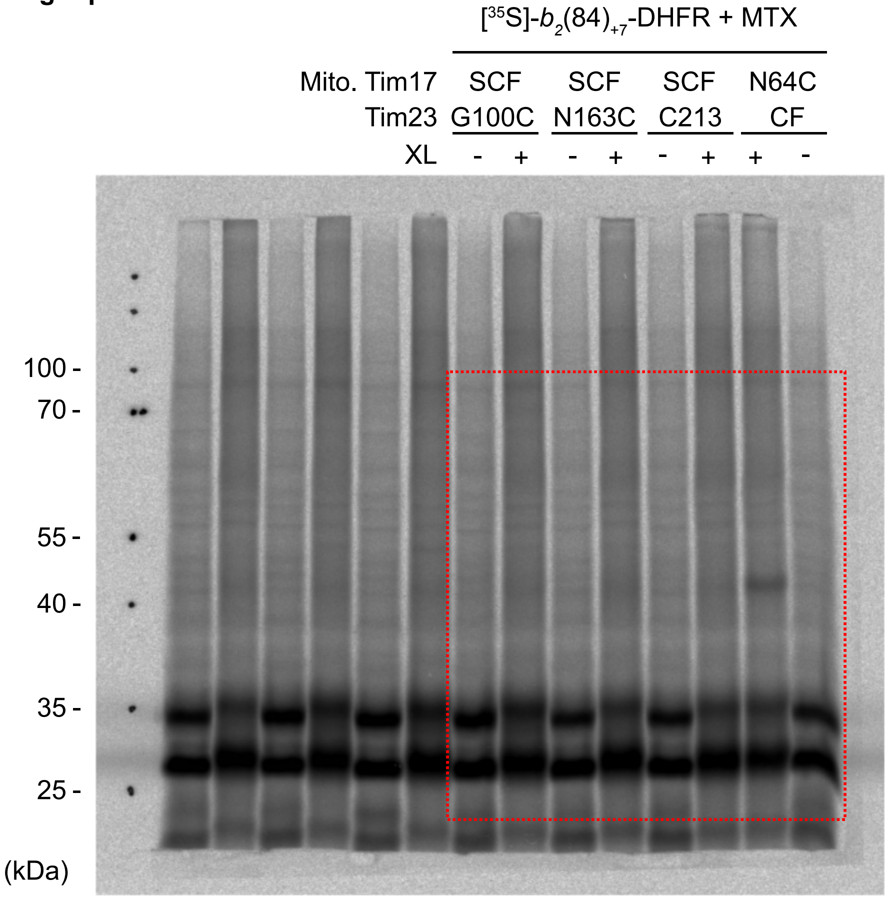

Extended data Figure 8d

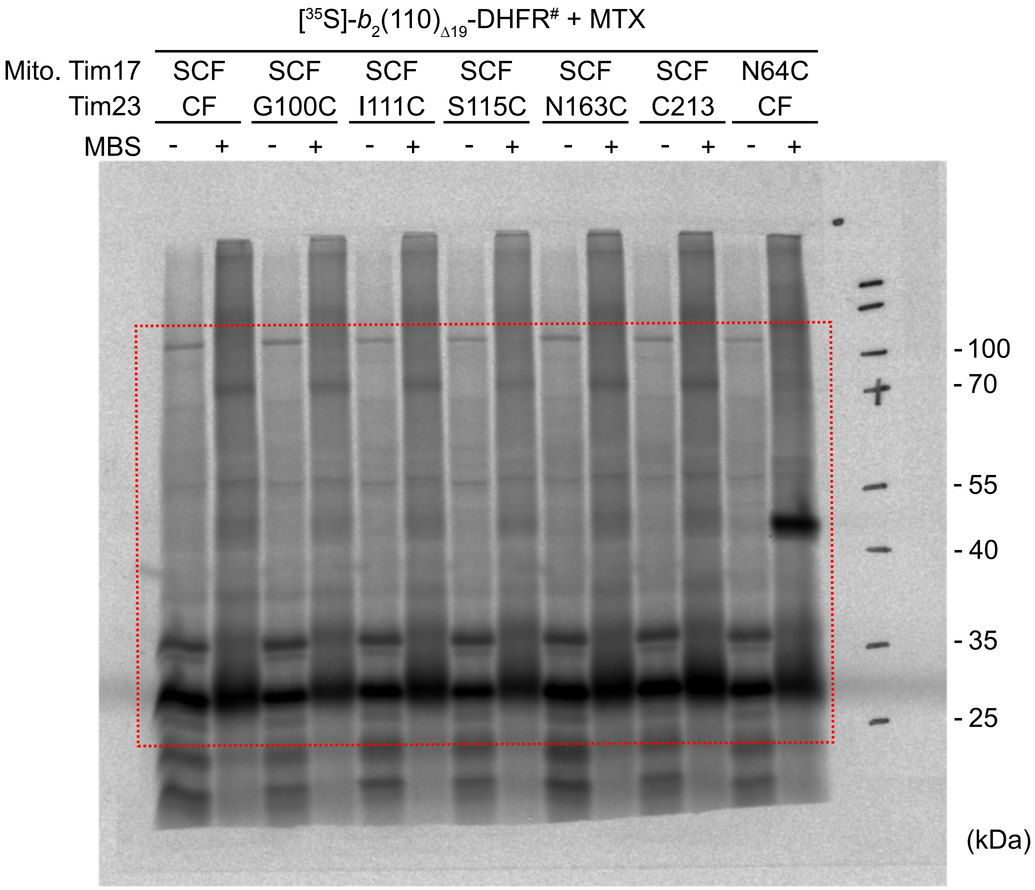

Extended data Figure 8e

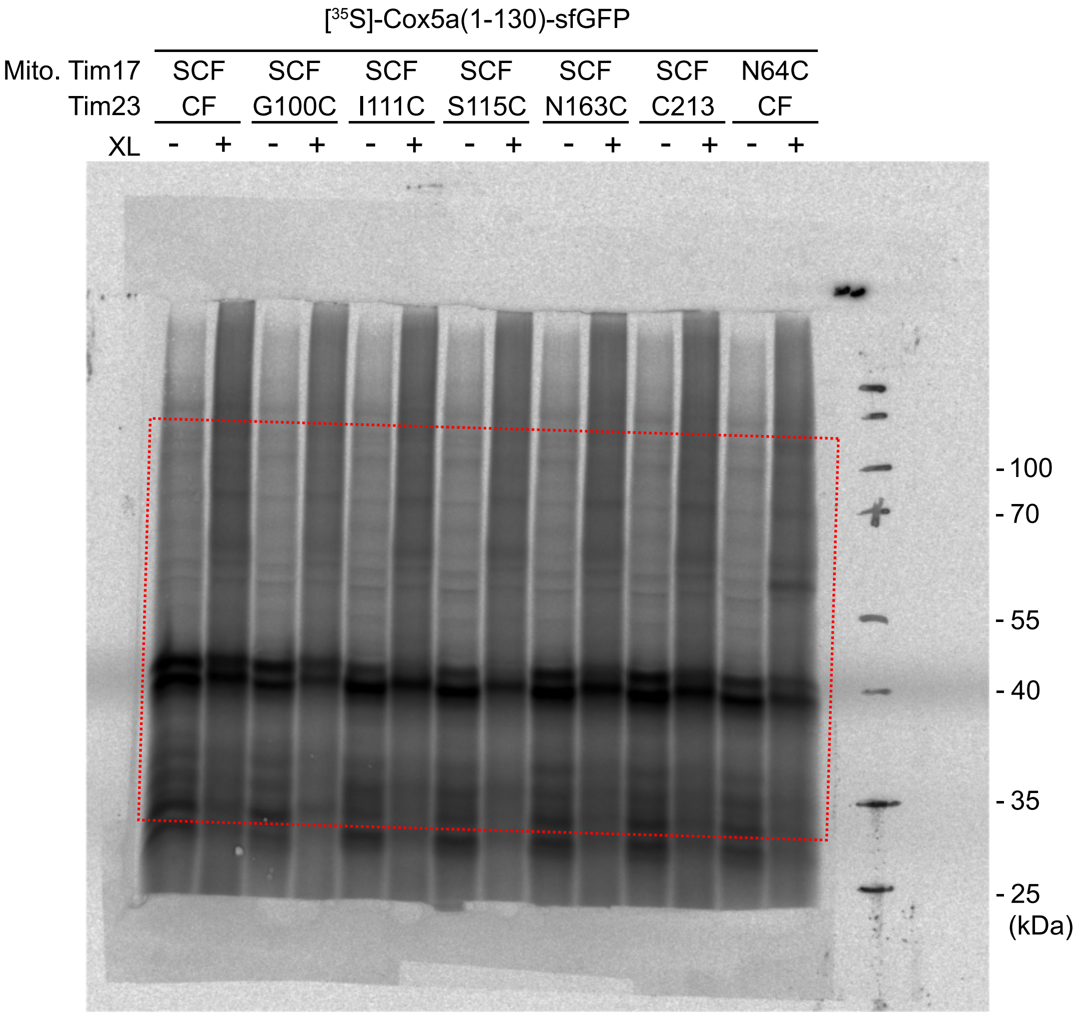

Extended Data Figure 8f

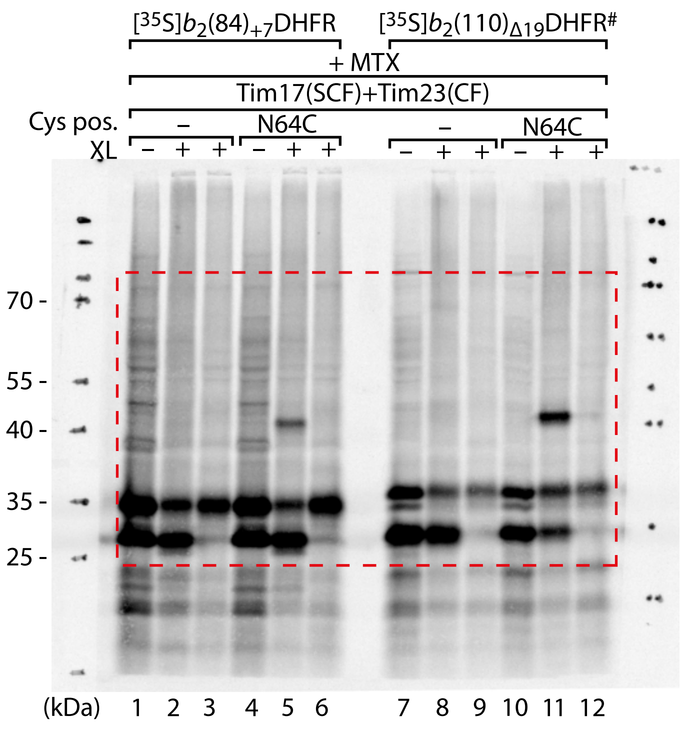

Extended Data Figure 8g

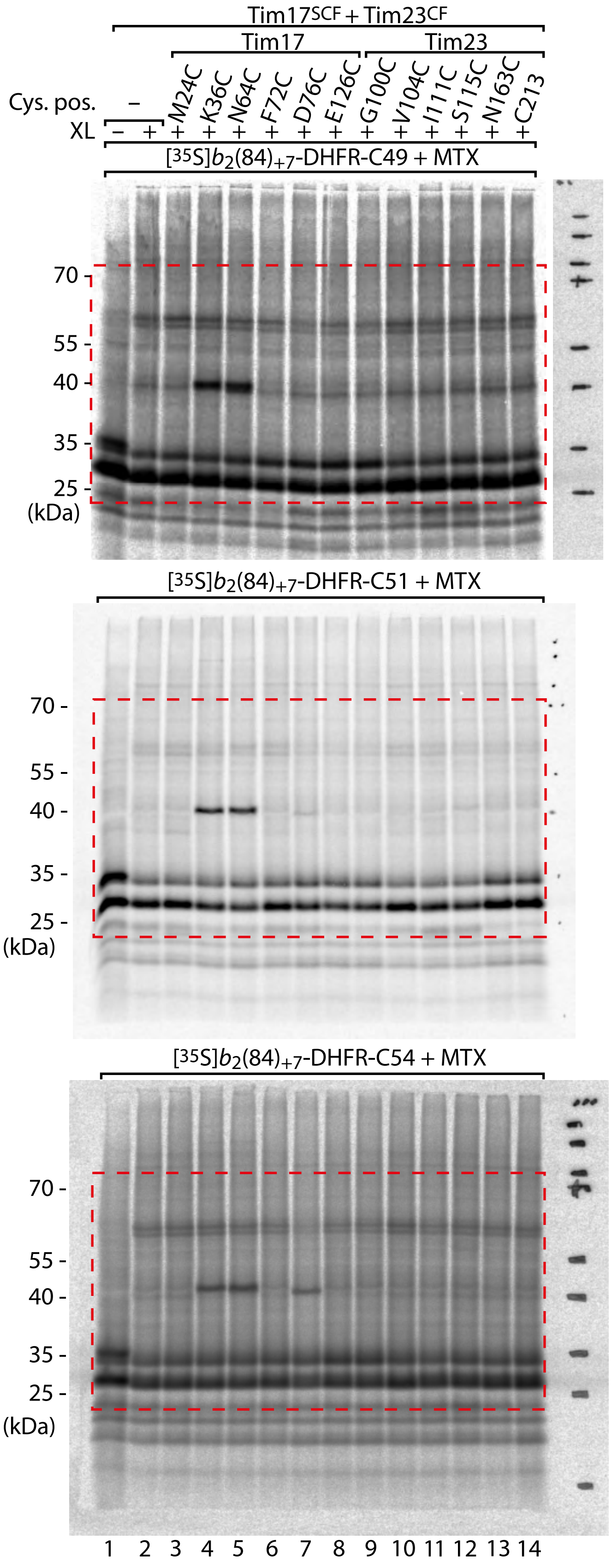

Extended Data Figure 8h

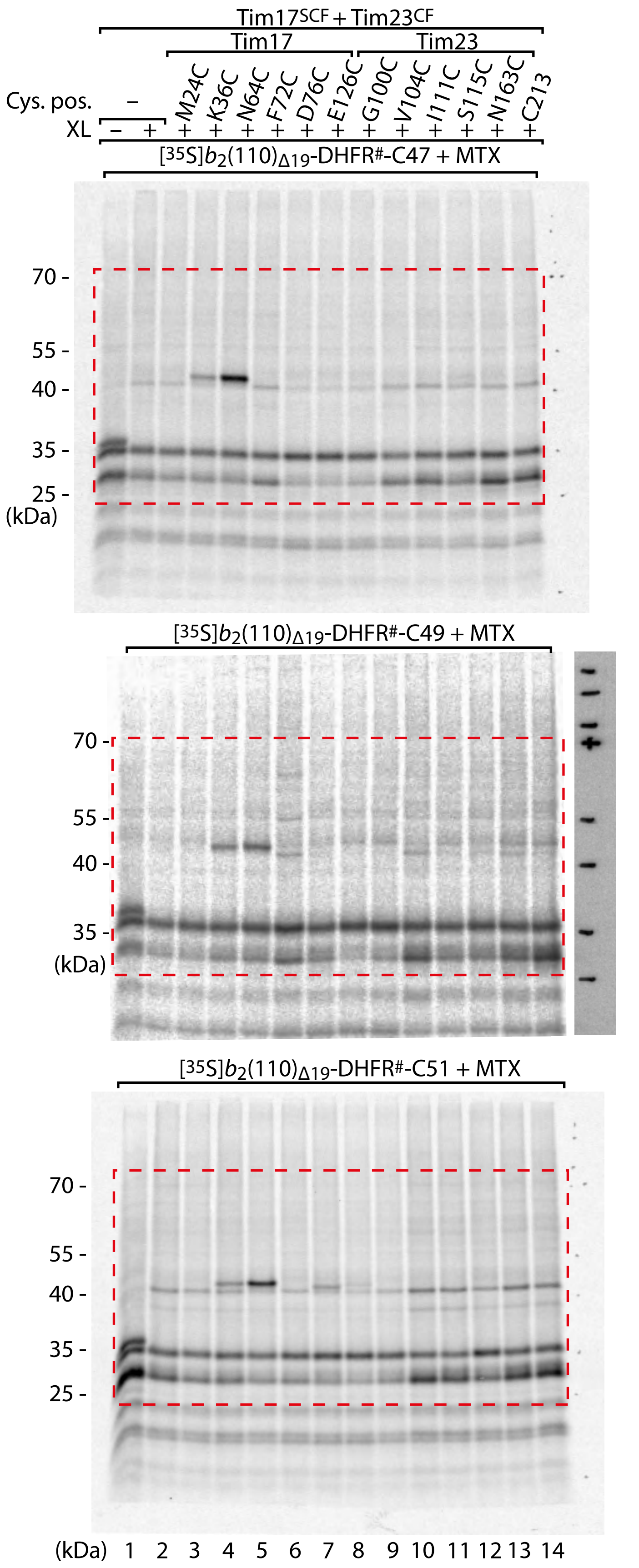

Supplementary Table 1. Yeast strains used in this study

Strain name	Source	Identifier
YPH499 *MATa ura3-52 lys2-801_amber ade2-101_ochre trp-*Δ*63 his3-*Δ*200 leu2-*Δ*1*	LGC Standards/ATCC	1501
YPH499 *tim17::ade2* [pYEp352-MET25pr-*TIM17*-CYC1t]	Ref. 21	-
YPH499 *tim23::ade2* [pYEp352-MET25pr-*TIM23*-CYC1t]	This study	-
YPH499 *tom22::his3* [pYEp352-MET25pr-*TOM22*-CYC1t]	Ref. 56	2281
YPH499 *tim17::hphNT1 tim23::ade2* [pYEp352-MET25pr-*TIM23*-CYC1t, pYEp352(HIS)-MET25pr-*TIM17*-CYC1t]	This study	-
YPH499 *tom22::his3* KanMX6GAL1-*TIM17* [pYEp352-MET25pr-*TOM22*-CYC1t]	This study	-
YPH499 *tim17::ade2* [pFL39-Tim17$_{D17A}$]	This study	-
YPH499 *tim17::ade2* [pFL39-Tim17$_{D17S}$]	This study	-
YPH499 *tim17::ade2* [pFL39-Tim17$_{D17L}$]	This study	-
YPH499 *tim17::ade2* [pFL39-Tim17$_{D17N}$]	This study	-
YPH499 *tim17::ade2* [pFL39-Tim17$_{D17T}$]	This study	-
YPH499 *tim17::ade2* [pFL39-Tim17$_{D17F}$]	This study	-
YPH499 *tim17::ade2* [pFL39-Tim17$_{D17R}$]	This study	-
YPH499 *tim17::ade2* [pFL39-Tim17$_{D76A}$]	This study	-
YPH499 *tim17::ade2* [pFL39-Tim17$_{D76S}$]	This study	-
YPH499 *tim17::ade2* [pFL39-Tim17$_{D76T}$]	This study	-
YPH499 *tim17::ade2* [pFL39-Tim17$_{D76L}$]	This study	-
YPH499 *tim17::ade2* [pFL39-Tim17$_{D76F}$]	This study	-
YPH499 *tim17::ade2* [pFL39-Tim17$_{D76N}$]	This study	-
YPH499 *tim17::ade2* [pFL39-Tim17$_{D76R}$]	This study	-
YPH499 *tim17::ade2* [pFL39-Tim17$_{E126A}$]	This study	-
YPH499 *tim17::ade2* [pFL39-Tim17$_{E126Q}$]	This study	-
YPH499 *tim17::ade2* [pFL39-Tim17$_{E126F}$]	This study	-
YPH499 *tim17::ade2* [pFL39-Tim17$_{E126R}$]	This study	-
YPH499 *tim17::ade2* [pFL39-Tim17$_{D17A_D76A}$]	This study	-
YPH499 *tim17::ade2* [pFL39-Tim17$_{D17A_E126A}$]	This study	-
YPH499 *tim17::ade2* [pFL39-Tim17$_{D76A_E126A}$]	This study	-
YPH499 *tim17::ade2* [pFL39-Tim17$_{N64L_S73L_T74L}$]	This study	-
YPH499 *tom22::his3* [pFL39-Tom22-Tim17]	This study	-
YPH499 *tom22::his3* [pFL39-Tom22-Tim17$_{Linker-2xStrep}$]	This study	-
YPH499 *tom22::his3* [pFL39-Tom22-$_{HisSUMO*}$Tim23]	This study	-
YPH499 *tom22::his3* KanMX6GAL1-*TIM17* [pFL39-Tom22]	This study	-
YPH499 *tom22::his3* KanMX6GAL1-*TIM17* [pFL39-Tom22-Tim17$_{WT}$]	This study	-
YPH499 *tom22::his3* KanMX6GAL1-*TIM17* [pFL39-Tom22-Tim17$_{D17A_D76A}$]	This study	-
YPH499 *tom22::his3* KanMX6GAL1-*TIM17* [pFL39-Tom22-Tim17$_{D17A_E126A}$]	This study	-
YPH499 *tom22::his3* KanMX6GAL1-*TIM17* [pFL39-Tom22-Tim17$_{D76A_E126A}$]	This study	-
YPH499 *tom22::his3* KanMX6GAL1-*TIM17* [pFL39-Tom22-Tim17$_{D17A_D76A_E126A}$]	This study	-
YPH499 *tom22::his3* KanMX6GAL1-*TIM17* *tim21::*Tim21$_{ProteinA-TEV-7His}$NatNT2 [pFL39-Tom22]	This study	-
YPH499 *tom22::his3* KanMX6GAL1-*TIM17* *tim21::*Tim21$_{ProteinA-TEV-7His}$NatNT2 [pFL39-Tom22-Tim17$_{D17A_D76A}$]	This study	-
YPH499 *tom22::his3* KanMX6GAL1-*TIM17* [pFL39-Tom22-Tim17$_{D17A_D76A}$]	This study	-
YPH499 *tim23::ade2 tim17::hphNT1* [pFL39-$_{HisSUMO*}$Tim23-Tim17$_{2xStrep}$]	This study	-
YPH499 *tim23::ade2 tim17::hphNT1* [pFL39-Tim23$_{CF}$-Tim17$_{SCF}$]	This study	-
YPH499 *tim23::ade2 tim17::hphNT1* [pFL39-Tim23$_{CF}$-Tim17$_{D17C}$]	This study	-
YPH499 *tim23::ade2 tim17::hphNT1* [pFL39-Tim23$_{CF}$-Tim17$_{M24C}$]	This study	-

YPH499 *tim23::ade2 tim17::hphNT1* [pFL39-Tim23$_{CF}$-Tim17$_{K36C}$]	This study	-
YPH499 *tim23::ade2 tim17::hphNT1* [pFL39-Tim23$_{CF}$-Tim17$_{N64C}$]	This study	-
YPH499 *tim23::ade2 tim17::hphNT1* [pFL39-Tim23$_{CF}$-Tim17$_{F72C}$]	This study	-
YPH499 *tim23::ade2 tim17::hphNT1* [pFL39-Tim23$_{CF}$-Tim17$_{D76C}$]	This study	-
YPH499 *tim23::ade2 tim17::hphNT1* [pFL39-Tim23$_{CF}$-Tim17$_{E126C}$]	This study	-
YPH499 *tim23::ade2 tim17::hphNT1* [pFL39-Tim23$_{G100C}$-Tim17$_{SCF}$]	This study	-
YPH499 *tim23::ade2 tim17::hphNT1* [pFL39-Tim23$_{V104C}$-Tim17$_{SCF}$]	This study	-
YPH499 *tim23::ade2 tim17::hphNT1* [pFL39-Tim23$_{I111C}$-Tim17$_{SCF}$]	This study	-
YPH499 *tim23::ade2 tim17::hphNT1* [pFL39-Tim23$_{S115C}$-Tim17$_{SCF}$]	This study	-
YPH499 *tim23::ade2 tim17::hphNT1* [pFL39-Tim23$_{N163C}$-Tim17$_{SCF}$]	This study	-
YPH499 *tim23::ade2 tim17::hphNT1* [pFL39-Tim23$_{C213}$-Tim17$_{SCF}$]	This study	-
YPH499 *tim23::ade2 tim17::hphNT1* [pFL39-Tim23$_{WT}$-Tim17$_{SCF_N16C}$]	This study	-
YPH499 *tim23::ade2 tim17::hphNT1* [pFL39-Tim23$_{I154C}$-Tim17$_{SCF_F22C}$]	This study	-
YPH499 *tim23::ade2 tim17::hphNT1* [pFL39-Tim23$_{L109C}$-Tim17$_{SCF_V67C}$]	This study	-

Supplementary Table 2. Plasmids used in this study

Plasmid	Source	Identifier
pGEM4Z-*b*$_2$(84)-DHFR	Ref. 48	B27
pGEM4Z-*b*$_2$(84)-DHFR_R47C	This study	-
pGEM4Z-*b*$_2$(84)-DHFR_K48C	This study	-
pGEM4Z-*b*$_2$(84)-DHFR_R49C	This study	-
pGEM4Z-*b*$_2$(84)-DHFR_T50C	This study	-
pGEM4Z-*b*$_2$(84)-DHFR_Q51C	This study	-
pGEM4Z-*b*$_2$(84)-DHFR_S52C	This study	-
pGEM4Z-*b*$_2$(84)-DHFR_W53C	This study	-
pGEM4Z-*b*$_2$(84)-DHFR_T54C	This study	-
pGEM4Z-*b*$_2$(110)$_\Delta$-DHFR	Ref. 14	2336
pGEM4Z-*b*$_2$(110)$_\Delta$-DHFR_C86S	This study	-
pGEM4Z-*b*$_2$(110)$_\Delta$-DHFR_S47C_C86S	This study	-
pGEM4Z-*b*$_2$(110)$_\Delta$-DHFR_S48C_C86S	This study	-
pGEM4Z-*b*$_2$(110)$_\Delta$-DHFR_V49C_C86S	This study	-
pGEM4Z-*b*$_2$(110)$_\Delta$-DHFR_A50C_C86S	This study	-
pGEM4Z-*b*$_2$(110)$_\Delta$-DHFR_Y51C_C86S	This study	-
pGEM4Z-*b*$_2$(110)$_\Delta$-DHFR_L52C_C86S	This study	-
pGEM4Z-*b*$_2$(110)$_\Delta$-DHFR_N53C_C86S	This study	-
pGEM4Z-*b*$_2$(110)$_\Delta$-DHFR_W54C_C86S	This study	-
pGEM4Z-*b*$_2$(167)$_\Delta$-DHFR	Ref. 48, 59	B04
pGEM4Z-*b*$_2$(167)-DHFR	Ref. 63	B03
pGEM4Z-$F_1\beta$	Wiedemann lab	F01
pGEM4Z-pSu9(1-69)-DHFR	Wiedemann lab	S02
pGEM4Z-*b*$_2$(220)$_\Delta$-DHFR	Wiedemann lab	B18
pGEM4Z-*b*$_2$(220)-DHFR	Ref. 14	B46
pGEM4Z-Cytochrome c_1	Wiedemann lab	C03
pGEM4Z-Aac2	Wiedemann lab	1039
pFL39-Tim17	Ref. 21	-
pFL39-Tom22	Ref. 56	1409
pFL39-Tom22-Tim17	This study	-
pFL39-Tim17$_{D17A}$	This study	-
pFL39-Tim17$_{D17S}$	This study	-
pFL39-Tim17$_{D17L}$	This study	-
pFL39-Tim17$_{D17N}$	This study	-
pFL39-Tim17$_{D17T}$	This study	-
pFL39-Tim17$_{D17F}$	This study	-
pFL39-Tim17$_{D17R}$	This study	-
pFL39-Tim17$_{D76A}$	This study	-
pFL39-Tim17$_{D76S}$	This study	-
pFL39-Tim17$_{D76T}$	This study	-

pFL39-Tim17$_{D76L}$	This study	-
pFL39-Tim17$_{D76F}$	This study	-
pFL39-Tim17$_{D76N}$	This study	-
pFL39-Tim17$_{D76R}$	This study	-
pFL39-Tim17$_{E126A}$	This study	-
pFL39-Tim17$_{E126Q}$	This study	-
pFL39-Tim17$_{E126F}$	This study	-
pFL39-Tim17$_{E126R}$	This study	-
pFL39-Tim17$_{D17A_D76A}$	This study	-
pFL39-Tim17$_{D17A_E126A}$	This study	-
pFL39-Tim17$_{D76A_E126A}$	This study	-
pFL39-Tim17$_{D17A_D76A_E126A}$	This study	-
pFL39-Tim17$_{C118G_C120V}$	This study	-
pFL39-Tim17$_{N16C_C118G_C120V}$	This study	-
pFL39-Tim17$_{L121C_C118G_C120V}$	This study	-
pFL39-Tim17$_{V124C_C118G_C120V}$	This study	-
pFL39-Tim17$_{N64L}$	This study	-
pFL39-Tim17$_{S114L}$	This study	-
pFL39-Tim17$_{M24D_G69D_C118E}$	This study	-
pFL39-Tim17$_{D17A_M24D_G69D_D76A_C118E_E126A}$	This study	-
pFL39-Tim17$_{G28D_F65D_S114E}$	This study	-
pFL39-Tim17$_{D17A_G28D_F65D_D76A_S114E_E126E}$	This study	-
pFL39-Tim17$_{D95A_D96A_D167A}$	This study	-
pFL39-Tim17$_{D95A_D96A_D167A_E221A}$	This study	-
pFL39-Tim17$_{D95A_D96A_D167A_G100D_N163D_C213D}$	This study	-
pFL39-Tom22-Tim17$_{D17A_D76A}$	This study	-
pFL39-Tom22-Tim17$_{D17A_E126A}$	This study	-
pFL39- Tom22-Tim17$_{D76A_E126A}$	This study	-
pFL39-Tom22-Tim17$_{D17A_D76A_E126A}$	This study	-
pFL39-Tom22-Tim17$_{GGGG-A-GGGG(Linker)-2xStrep}$	This study	-
pFL39-Tom22- $_{HisSumo*}$Tim23	This study	-
pFL39-Tim23-Tim17	This study	-
pFL39-$_{HisSumo*}$Tim23-Tim17$_{Linker-2xStrep}$	This study	-
pFL39-Tim23$_{G100D_N163D_C213D}$-Tim17$_{D17A_D76A_E126A}$	This study	-
pFL39-Tim23$_{CF}$-Tim17$_{SCF}$	This study	-
pFL39-Tim23$_{CF}$-Tim17$_{SCF_D17C}$	This study	-
pFL39-Tim23$_{CF}$-Tim17$_{SCF_M24C}$	This study	-
pFL39-Tim23$_{CF}$-Tim17$_{SCF_K36C}$	This study	-
pFL39-Tim23$_{CF}$-Tim17$_{SCF_N64C}$	This study	-
pFL39-Tim23$_{CF}$-Tim17$_{SCF_F72C}$	This study	-
pFL39-Tim23$_{CF}$-Tim17$_{SCF_D76C}$	This study	-
pFL39-Tim23$_{CF}$-Tim17$_{SCF_E126C}$	This study	-

pFL39-Tim23$_{CF_G100C}$ -Tim17$_{SCF}$	This study	-
pFL39-Tim23$_{CF_V104C}$ -Tim17$_{SCF}$	This study	-
pFL39-Tim23$_{CF_I111C}$ -Tim17$_{SCF}$	This study	-
pFL39-Tim23$_{CF_S115C}$ -Tim17$_{SCF}$	This study	-
pFL39-Tim23$_{CF_N163C}$ -Tim17$_{SCF}$	This study	-
pFL39-Tim23$_{CF_C213}$ -Tim17$_{SCF}$	This study	-
pFL39-Tim23$_{WT}$ -Tim17$_{SCF_N16C}$	This study	-
pFL39-Tim23$_{CF_I154C}$ -Tim17$_{SCF_F22C}$	This study	-
pFL39-Tim23$_{CF_L109C}$ -Tim17$_{SCF_V67C}$	This study	-
pSUMO-His$_{14}$-SUMO-Jac1-sfGFP	Ref. 20	-
pSUMO-His$_{14}$-SUMO-Cox5a(1-130)-sfGFP	This study	-
pFA6a-hphNT1	Ref. 64	
pFA6a-kanMX6-PGAL1	Ref. 57	1437
pFA6a-NatNT1-Protein A-TEV-7xHis	This study	2724

Supplementary Table 3. Primers used in this study

Primer name	Sequence 5' → 3'
SP6 Tim17 fw	GATCGATTTAGGTGACACTATAGAAGCGGCCACCATGTCAGCCGATCA TTCGAGAGATCC
Tim17 rev TAA	CGTACGGATCCTTAAGCTTGCAGAGGTTGAGAGG
SP6-Oxa1	TCGATTTAGGTGACACTATAGAATACGCCGCCGCCATGTTCAAACTCAC CTCTCGAC
Oxa1 rev	GATCTCATTTTTTGTTATTAATGAAGTTTG
WT DIC_SP6_Fwd	TCGATTTAGGTGACACTATAGAATACGCCGCCGCCATGTCAACCAACG CAAAAGAG
DIC WT_Rev	CTACTTGTCTTCCTTTGGCATG
SP6-Cox5a_Fwd	TCGATTTAGGTGACACTATAGAATACGCCGCCGCCATGTTACGTA
sfGFP_Rev	TTATTTATACAGCTCGTCCATGCC
EcoRI_Tom22p_Fwd	CAGTCGAATTCGGCTGAAGATATCTATAG
EcoRI_Tom22t_Rev	GACTGGAATTCCGCATCGGAAGTTCATAGAAG
EcoRI_Tim17p_Fwd	CAGTCGAATTCCGATGAAAACGGCAG
EcoRI_Tim17t_Rev	GACTGGAATTCAAGGAAGGAGAATACATCTGGC
EcoRI_Tim23p_Fwd	CAGTCGAATTCCAAGTATAAGTGTCATG
EcoRI_Tim23t_Rev	GACTGGAATTCCTTTAATTGGCCATCGAAAACAATAG
Tim17_D17A fw	CATACTAAATGCGTTCGGTGGTGCTTTTG
Tim17_D17A rev	CACCACCGAACGCATTTAGTATGACTATAG
Tim17_D76A_fw	GACTTTTGCGTGCGCTGTGAAGGCCGTTAG
Tim17_D76A_rev	CACAGCGCACGCAAAAGTCGAAAATAAAC
Tim17_E126A_fw	GTGTGATTGCAGGTGTGGGACTAATG
Tim17_E126A_rev	CCACACCTGCAATCACACCCAAC
Tim17_D17R_fwd	CATACTAAATAGATTCGGTGGTGCTTTTG
Tim17_D17R_rev	CCACCGAATCTATTTAGTATGACTATAG
Tim17_D17N_fwd	CATACTAAATAATTTCGGTGGTGC
Tim17_D17N_rev	CACCGAAATTATTTAGTATGACTATAG
Tim17_D17F_fwd	CATACTAAATTTTTTCGGTGGTGCTTTTG
Tim17_D17F_rev	CCACCGAAAAAATTTAGTATGACTATAG
Tim17_D76R_fwd	CGACTTTTAGATGCGCTGTGAAGG
Tim17_D76R_rev	CACAGCGCATCTAAAAGTCGAAAATAAAC
Tim17_D76N_fwd	CGACTTTTAATTGCGCTGTGAAG
Tim17_D76N_rev	CAGCGCAATTAAAAGTCGAAAATAAAC
Tim17_D76F_fwd	CGACTTTTTTTTGCGCTGTGAAG
Tim17_D76F_rev	CAGCGCAAAAAAAAGTCGAAAATAAAC
Tim17_E126R_fwd	GTGTGATTAGAGGTGTGGGACTAATG
Tim17_E126R_rev	CCACACCTCTAATCACACCCAAC
Tim17_E126Q_fwd	GTGTGATTCAAGGTGTGGGACTAATG
Tim17_E126Q_rev	CACACCTTGAATCACACCCAAC

Tim17_E126F_fwd	GTGTGATTTTTGGTGTGGGACTAATG
Tim17_E126F_rev	CCCACACCAAAAATCACACCCAAC
Tim17_D17T_fwd	CATACTAAATACTTTCGGTGGTGCTTTTG
Tim17_D17T_rev	CCACCGAAAGTATTTAGTATGACTATAGG
Tim17_D17S_fwd	CATACTAAATTCTTTCGGTGGTGCTTTTG
Tim17_D17S_rev	CCACCGAAAGAATTTAGTATGACTATAG
Tim17_D17L_fwd	CATACTAAATTTGTTCGGTGGTGCTTTTG
Tim17_D17L_rev	CCACCGAACAAATTTAGTATGACTATAGG
Tim17_D76T_fwd	CGACTTTTACTTGCGCTGTGAAG
Tim17_D76T_rev	CAGCGCAAGTAAAAGTCGAAAATAAAC
Tim17_D76S_fwd	CGACTTTTTCTTGCGCTGTGAAG
Tim17_D76S_rev	CAGCGCAAGAAAAAGTCGAAAATAAAC
Tim17_D76L_fwd	CGACTTTTTTGTGCGCTGTGAAGG
Tim17_D76L_rev	CACAGCGCACAAAAAAGTCGAAAATAAAC
Tim17_C118G_C120V_fwd	CTGTTTTGTTGGGTGTGATTGAAGGTGTG
Tim17_C118G_C120V_rev	CACCCGTGATCGAACTGTTCCTTGTATG
Tim17_N16C_fwd	GTCATACTATGTGATTTCGGTGGTG
Tim17_N16C_rev	CCGAAATCACATAGTATGACTATAGG
Tim17_C118G_C120V_L121C_fwd	GTGCTGTTTGTTTGGGTGTGATTG
Tim17_C118G_C120V_L121C_rev	CACACCCAAACAAACAGCACCCGTG
Tim17_C118G_C120V_V124C_fwd	GTTGGGTTGTATTGAAGGTGTGG
Tim17_C118G_C120V_V124C_rev	CCTTCAATACAACCCAACAAAACAG
Tim17_N64L_fwd	CTGGGTGGTTTGTTTGGTGTGTGGG
Tim17_N64L_rev	CACACCAAACAAACCACCCAGTACG
Tim17_S114L_fwd	CAAGGAACTTATCGATCACGTGTGCTTG
Tim17_S114L_rev	CGTGATCGATAAGTTCCTTGTATGCCTC
pGal-Tim17_F4	ACTCCAGCATTATAAAGCATATCTAACAATACCATTCGGGTTATACTGAATAGCCGAATTCGAGCTCGTTTAAAC
pGal-Tim17_R2	AAATCATTTAGTATGACTATAGGACATGGATCTCTCGAATGATCGGCTGACATTTTGAGATCCGGGTTTT

Tim17_D17C_fwd	TGTTTCGGTGGTGCTTTTGCCATG
Tim17_D17C_rev	ATTTAGTATGACTATAGGACATGGATC
Tim17_M24C_fwd	TGTGGTGCCATTGGTGGTGTTG
Tim17_M24C_rev	GGCAAAAGCACCACCGAAATC
Tim17_K36C_fwd	TGTGGTTTTAGAAATTCGCCATTAGGTG
Tim17_K36C_rev	AATCCCATGCCAAACAACACC
Tim17_N64C_fwd	TGTTTTGGTGTGTGGGGTGGTTTA
Tim17_N64C_rev	ACCACCCAGTACGGGAG
Tim17_F72C_fwd	TGTTCGACTTTTGATTGCGCTGTG
Tim17_F72C_rev	TAAACCACCCCACACACC
Tim17_D76C_fwd	TGTTGCGCTGTGAAGGCCG
Tim17_D76C_rev	AAAAGTCGAAAATAAACCACCCC
Tim17_E126C_fwd	TGTGGTGTGGGACTAATGTTTCAAAG
Tim17_E126C_rev	AATCACACCCAACAAAACAGCAC
Tim17_F22C_fwd	TGTGCCATGGGTGCCATTGG
Tim17_F22C_rev	AGCACCACCGAAATCATTTAGTAT
Tim17_V67C_fwd	TGCTGGGGTGGTTTATTTTCGACTTTTG
Tim17_V67C_rev	ACCAAAATTACCACCCAGTACG
Tim17_M24D_Fwd	CTTTTGCCGATGGTGCCATTGGTG
Tim17_M24D_Rev	CAATGGCACCATCGGCAAAAGCAC
Tim17_G69D_Fwd	GTGTGTGGGATGGTTTATTTTCGAC
Tim17_G69D_Rev	GAAAATAAACCATCCCACACACCAAAATTAC
Tim17_C118E_Fwd	CGATCACGGAAGCTTGTTTGTTGG
Tim17_C118E _Rev	CAAACAAGCTTCCGTGATCGAACTG
Tim17_G28D_Fwd	GTGCCATTGATGGTGTTGTTTGG
Tim17_G28D_Rev	CAACACCATCAATGGCACCCATG
Tim17_F65D_Fwd	GTGGTAATGATGGTGTGTGGGGTG
Tim17_F65D_Rev	CACACACCATCATTACCACCCAGTAC
Tim17_S114E_Fwd	CAAGGAACGAATCGATCACGTGTG
Tim17_S114E_Rev	GTGATCGATTCGTTCCTTGTATGC
Tim23_G100C_fwd	TGTACCGGTGCCGTCTACCTG
Tim23_G100C_rev	GTACAATAGGTCATCGGTCCAC
Tim23_V104C_fwd	TGTTACCTGCTGGGACTTGGTATCG
Tim23_V104C_rev	GGCACCGGTACCGTACAATAG
Tim23_I111C_fwd	TGTGGAGGGTTTTCTGGTATGATG

Tim23_I111C_rev	ACCAAGTCCCAGCAGGTAG
Tim23_S115C_fwd	TGTGGTATGATGCAGGGTCTGC
Tim23_S115C_rev	AAACCCTCCGATACCAAGTCC
Tim23_N163C_fwd	TGTTCTACAATAGATGCACTAAGAGG
Tim23_N163C_rev	GATGATATTGTAGCTCAACGCG
Tim23_A213C_fwd	TGTAGTGTCAAGAAAAGACTACTTGAAAAATG
Tim23_A213C_rev	CCAGACGGCAGCCGCAG
Tim23_I154C_fwd	TGTCTCGCGTTGAGCTACAATATCATC
Tim23_I154C_rev	CCCCGCATTATTACCTAAGAAGG
Tim23_L109C_fwd	TGTGGTATCGGAGGGTTTTCTGG
Tim23_L109C_rev	TCCCAGCAGGTAGACGGC
Tim23_D95A D96A_Fwd	GTGGACCGCTGCCCTATGTTACGGTAC
Tim23_D95A D96A_Rev	GTAACATAGGGCAGCGGTCCACCCACG
Tim23_D167A_Fwd	CTACAATAGCTGCACTAAGAGGCAAAC
Tim23_D167A_Rev	CTTAGTGCAGCTATTGTAGAATTG
Tim23_E221A_Fwd	GACTACTTGCAAAATGAGCAACACAAG
Tim23_E221A_Rev	GTTGCTCATTTTGCAAGTAGTCTTTTC
Tim23_G100D_Fwd	CTATGTTACGATACCGGTGCCGTC
Tim23_G100D_Rev	CACCGGTATCGTAACATAGGTCATC
Tim23_N163D_Fwd	CAATATCATCGATTCTACAATAGATG
Tim23_N163D_Rev	GTAGAATCGATGATATTGTAGCTC
Tim23_C213D_Fwd	GCCGTCTGGGATAGTGTCAAGAAAAGAC
Tim23_C213D_Rev	CTTGACACTATCCCAGACGGCGCAC
b2-110d19-C86S for	ATATTGATCACAACCCAACTATCATCGGGCTTGTTATGC
b2-110d19-C86S rev	GCATAACAAGCCCGATGATAGTTGGGTTGTGATCAATAT
b2-84-R47C for	GTCGTTCGAACAAGACTCATGCAAACGCACACAGTCATGGA
b2-84-R47C rev	TCCATGACTGTGTGCGTTTGCATGAGTCTTGTTCGAACGAC
b2-84-K48C for	AAGTCGTTCGAACAAGACTCAAGATGCCGCACACAGTCATGGACTG
b2-84-K48C rev	CAGTCCATGACTGTGTGCGGCATCTTGAGTCTTGTTCGAACGACTT
b2-84-R49C for	GTCGTTCGAACAAGACTCAAGAAAATGCACACAGTCATG
b2-84-R49C rev	CATGACTGTGTGCATTTTCTTGAGTCTTGTTCGAACGAC
b2-84-T50C for	GAACAAGACTCAAGAAAACGCTGCCAGTCATGGACTGCCTTGAGA
b2-84-T50C rev	TCTCAAGGCAGTCCATGACTGGCAGCGTTTTCTTGAGTCTTGTTC
b2-84-Q51C for	CGTTCGAACAAGACTCAAGAAAACGCACATGCTCATGGACTGCCTTG

b2-84-Q51C rev	CAAGGCAGTCCATGAGCATGTGCGTTTTCTTGAGTCTTGTTCGAACG
b2-84-S52C for	CAAGACTCAAGAAAACGCACACAGTGCTGGACTGCCTTGA
b2-84-S52C rev	TCAAGGCAGTCCAGCACTGTGTGCGTTTTCTTGAGTCTTG
b2-84-W53C for	ACGCACACAGTCATGCACTGCCTTGAGAGTC
b2-84-W53C rev	GACTCTCAAGGCAGTGCATGACTGTGTGCGT
b2-84-T54C for	CGCACACAGTCATGGTGTGCCTTGAGAGTCGG
b2-84-T54C rev	CCGACTCTCAAGGCACACCATGACTGTGTGCG
b2-110d19-S47C for	CGTTCGAACAAGACTCATGTTCCGTGGCGTATCTA
b2-110d19-S47C rev	TAGATACGCCACGGAACATGAGTCTTGTTCGAACG
b2-110d19-S48C for	CGTTCGAACAAGACTCAAGTTGCGTGGCGTATCTA
b2-110d19-S48C rev	TAGATACGCCACGCAACTTGAGTCTTGTTCGAACG
b2-110d19-V49C for	GTTCGAACAAGACTCAAGTTCCTGCGCGTATCTAAACTGGCATAATG
b2-110d19-V49C rev	CATTATGCCAGTTTAGATACGCGCAGGAACTTGAGTCTTGTTCGAAC
b2-110d19-A50C for	GAACAAGACTCAAGTTCCGTGTGCTATCTAAACTGGCATAATGGC
b2-110d19-A50C rev	GCCATTATGCCAGTTTAGATAGCACACGGAACTTGAGTCTTGTTC
b2-110d19-Y51C for	GACTCAAGTTCCGTGGCGTGTCTAAACTGGCATA
b2-110d19-Y51C rev	TATGCCAGTTTAGACACGCCACGGAACTTGAGTC
b2-110d19-L52C for	AAGACTCAAGTTCCGTGGCGTATTGCAACTGGCATAATGGCCAAATAG
b2-110d19-L52C rev	CTATTTGGCCATTATGCCAGTTGCAATACGCCACGGAACTTGAGTCTT
b2-110d19-N53C for	ACTCAAGTTCCGTGGCGTATCTATGCTGGCATAATGGCC
b2-110d19-N53C rev	GGCCATTATGCCAGCATAGATACGCCACGGAACTTGAGT
b2-110d19-W54C for	CGTGGCGTATCTAAACTGCCATAATGGCCAAATAGAC
b2-110d19-W54C rev	GTCTATTTGGCCATTATGGCAGTTTAGATACGCCACG
Cox5a(1-130)_Fwd	TGGCGAGCTCATGTTACGTAACACTTTTACTAG
Cox5a(1-130)_Rev	TACCCTGCAGTAGCTGCCACTCCTTATTC
sfGFP_Fwd	GTGGCAGCTACTGCAGGGTAAAGGCGAAG
pSUMO_Rev	TACGTAACATGAGCTCGCCACCAATCTG

Supplementary Table 4. Antibodies used in this study

Antibodies	Source	Identifier	Dilution
Tim17	Ref. 65	GR1844-6, GR1844-7	1:500; 1:200 (AP)
Tim21	Ref. 65	GR3883-7	1:200
Tim23	Ref. 65	GR3878-6, 133-14	1:200 to 1:500
Tim44	Ref. 65	GR1835-4, 127-5, 127-6	1:200 to 1:500
Tim50	Ref. 65	GR3881-1, GR5185-4	1:500 to 1:1,000
Mgr2	Ref. 65	GR3120-1	1:100 (AP)
Aco1	Ref. 65	GR945-3, GR945-4	1:500
Atp1	Ref. 66	GR 5075-4	1:1,000
Cox1	Ref. 65	GR1538-13, GR1539-3	1:500
Cox4	Ref. 65	GR578-4	1:500
Cox12	Ref. 67	GR1937-4	1:250
Mdh1	Ref.68	GR1088-6	1:1,000
mtHsp70/Ssc1	Ref. 67	GR1830-3, GR1830-7	1:1,000
Tim22	Ref. 69	GR5113-4	1:250
Tom5	Ref. 70	GR3420-7	1:500
Tom22	Ref. 65	GR3227-2, GR3225-7	1:500
Tom40	Ref. 65	168-4, 168-13	1:500
Tom70	Ref. 65	GR657-3	1:250 to 1:500
Yme1	Ref. 65	GR1435-6	1:500
Qcr8	Ref. 65	GR1037-5	1:300
GFP	Roche	#11814460001, Lot: 65309400	1:1,000
Goat anti-rabbit-HRP	Jackson ImmunoResearch Laboratories	#111-035-003, Lot: 162282	1:5,000 to 1:20,000
Goat anti-rabbit IgG-HRP	Sigma-Aldrich	#A6154, Lot: SLBG72001V	1:8,000 to 1:10,000
Horse anti-mouse-HRP	Cell Signaling Technology	#7076S, Lot: 38	1:2,000
