# Supplementary material for: Central role of Tim17 in mitochondrial presequence protein translocation
Source: Nature. 2023 Aug 1;621(7979):627–34. doi: 10.1038/s41586-023-06477-8 (PMC10511324; doi:10.1038/s41586-023-06477-8)
Supplement: Supplementary file 2 — Reporting Summary [file 41586_2023_6477_MOESM2_ESM.pdf]

nature portfolio

Corresponding author(s): Martin van der Laan
Nils Wiedmann

Last updated by author(s): Aug 15, 2023

Reporting Summary

Nature Portfolio wishes to improve the reproducibility of the work that we publish. This form provides structure for consistency and transparency in reporting. For further information on Nature Portfolio policies, see our Editorial Policies and the Editorial Policy Checklist.

Statistics

For all statistical analyses, confirm that the following items are present in the figure legend, table legend, main text, or Methods section.

n/a	Confirmed	
☒	☐	The exact sample size (*n*) for each experimental group/condition, given as a discrete number and unit of measurement
☒	☐	A statement on whether measurements were taken from distinct samples or whether the same sample was measured repeatedly
☒	☐	The statistical test(s) used AND whether they are one- or two-sided<br>*Only common tests should be described solely by name; describe more complex techniques in the Methods section.*
☒	☐	A description of all covariates tested
☒	☐	A description of any assumptions or corrections, such as tests of normality and adjustment for multiple comparisons
☒	☐	A full description of the statistical parameters including central tendency (e.g. means) or other basic estimates (e.g. regression coefficient) AND variation (e.g. standard deviation) or associated estimates of uncertainty (e.g. confidence intervals)
☒	☐	For null hypothesis testing, the test statistic (e.g. *F*, *t*, *r*) with confidence intervals, effect sizes, degrees of freedom and *P* value noted<br>*Give P values as exact values whenever suitable.*
☒	☐	For Bayesian analysis, information on the choice of priors and Markov chain Monte Carlo settings
☒	☐	For hierarchical and complex designs, identification of the appropriate level for tests and full reporting of outcomes
☒	☐	Estimates of effect sizes (e.g. Cohen's *d*, Pearson's *r*), indicating how they were calculated

Our web collection on statistics for biologists contains articles on many of the points above.

Software and code

Policy information about availability of computer code

Data collection	Typhoon FLA-7000 ver. 1.2 (build 1.2.1.93), Image Reader FLA-7000 ver. 1.12 for autoradiography; Image Reader LAS-3000 ver. 2.21, Image Reader LAS-4000 ver. 2.1, SilverFast (MicrotekSDK) ver. 6.6.1r7 for immunodecoration signals; AB2 Luminescence Spectrophotometer ver. 5.50 (Thermo Electron) for assessment of the mitochondrial membrane potential; CLARIOstar ver. 5.61 (BMG LABTECH) for monitoring yeast growth; Nanodrop ND-1000 ver. 3.5.2. (Coleman Technologies Inc. for Nanodrop Tech.) for spectroscopy; Unicorn ver. 7.2. (General Electric Company) for usage of the Äkta pureTM system.
Data analysis	PyMOL ver. 2.4.1 for introducing other amino acid residues to the structure of Tim17 and Tim23; Affinity Photo ver. 1.10.6., ColabFold ver. 1.5.2, Geneious Prime ver. 2022.1.1, GraphPad Prism ver. 9.0.0 (121), ImageJ ver. 1.49v, Jalview ver. 2.11.2.7., Multi Gauge ver. 3.0 (Fujifilm), SnapGene viewer ver. 6.2.1, Adobe Illustrator 2023 ver. 27.6., Affinity Designer ver. 1.10.6., UCSF ChimeraX ver. 1.6.1., CLARIOstar data analysis MARS ver. 3.41 (BMG LABTECH) for processing and figure preparation.

For manuscripts utilizing custom algorithms or software that are central to the research but not yet described in published literature, software must be made available to editors and reviewers. We strongly encourage code deposition in a community repository (e.g. GitHub). See the Nature Portfolio guidelines for submitting code & software for further information.

Data

Policy information about availability of data

All manuscripts must include a data availability statement. This statement should provide the following information, where applicable:

- Accession codes, unique identifiers, or web links for publicly available datasets
- A description of any restrictions on data availability
- For clinical datasets or third party data, please ensure that the statement adheres to our policy

We employed the following publicly available data: Tim22 structures from (PDB ID 6LO8 and PDB ID 7CGP; https://www.rcsb.org); AlphaFold structural models (https://alphafold.ebi.ac.uk) of Tim17 (AF-P39515-F1) and Tim23 (AF-P32897-F1); predicted interaction between Tim23 and Tim17 (doi:10.5452/ma-bak-cepc-0314) and between Tim17 and Mgr2 (doi:10.5452/ma-bak-cepc-0515). Uncropped gels, blots and source data are shown in Supplementary Fig. 1 and Supplementary Table 5.

Human research participants

Policy information about studies involving human research participants and Sex and Gender in Research.

Reporting on sex and gender	N/A
Population characteristics	N/A
Recruitment	N/A
Ethics oversight	N/A

Note that full information on the approval of the study protocol must also be provided in the manuscript.

Field-specific reporting

Please select the one below that is the best fit for your research. If you are not sure, read the appropriate sections before making your selection.

☒ Life sciences ☐ Behavioural & social sciences ☐ Ecological, evolutionary & environmental sciences

For a reference copy of the document with all sections, see nature.com/documents/nr-reporting-summary-flat.pdf

Life sciences study design

All studies must disclose on these points even when the disclosure is negative.

Sample size	Sample sizes used for biochemical experiments were selected based on previous experiences with specific types of experiments like the amount of mitochondria used for protein level analysis, in vitro import and pulldown experiments (Ieva et al., 2014, doi:10.1016/j.molcel.2014.10.010; Weinhäupl et al., 2018, doi:10.1016/j.cell.2018.10.039; Höhr et al., 2018, doi:10.1126/science.aah6834; Takeda et al., 2021, doi:10.1038/s41586-020-03113-7). According to this the required amount of yeast cells or mitochondria was selected for each experiment. For experiments with modifications compared to previous types, several runs were performed to determine an optimal sample size. The sample sizes for the experiments are stated in the Methods section.
Data exclusions	All relevant data shown. No data were excluded from the analysis.
Replication	Representative images are shown for in vitro imports of radiolabelled precursor proteins into isolated mitochondria, chemical crosslinking, blue native electrophoresis, in vitro oxidation and reduction, yeast growth (wild-type and mutants), protein level analysis and affinity purifications by immunodecoration, assessment of the mitochondrial membrane potential of isolated mitochondria. The findings were confirmed by independent experiments (minimum number of independent experiments in parentheses) for the following figures 1c (2), 1d (3), 1f (3), 1g (3), 2b (2), 2c (2), 2d (2), 2e (2), 3b (2), 3c (3), 4a (2), 4b (2), 4c (2), 4d (2), 4e (2), 5b (2), 5c (2), 5d (2) and Extended Data figures ED1a (2), ED1b (2), ED1c (2), ED1d (2), ED2b (2), ED2c (2), ED2d (2), ED2e (2), ED2f (3), ED3c (2), ED3d (2), ED3f (3), ED5c (2), ED6a (2), ED6b (2), ED6c (2), ED6d (2), ED6e (2), ED6f (2), ED6g (2), ED6h (2), ED6i (2), ED7b (3), ED7c (3), ED7d (2), ED7e (2), ED7f (2), ED7g (3), ED7h (2), ED7i (2), ED8a (2), ED8b (2), ED8c (2), ED8d (2), ED8e (2), ED8f (2), ED8g (2), ED8h (2). The crucial experiments with phenotypes of Tim17 charge and hydrophilic mutants include biological replicates using different mitochondrial preparations (Fig. 1f-g; Fig. 3b-c; Fig. 4b-c; Extended data Fig. 2c, e-f; Extended data Fig. 6b; Extended data Fig. 7c-e and i), the other experiments represent technical replicates.
Randomization	All yeast clones for growth tests, mitochondrial isolations and biochemical experiments were selected randomly. The experiments employing isolated mitochondria were not randomized. All samples in one experiment were processed in parallel with the same buffers and under the same conditions.
Blinding	Blinding was not performed. The yeast strains had to be validated before they were used for experiments.

Reporting for specific materials, systems and methods

We require information from authors about some types of materials, experimental systems and methods used in many studies. Here, indicate whether each material, system or method listed is relevant to your study. If you are not sure if a list item applies to your research, read the appropriate section before selecting a response.

Materials & experimental systems

n/a	Involved in the study
☐	☒ Antibodies
☒	☐ Eukaryotic cell lines
☒	☐ Palaeontology and archaeology
☒	☐ Animals and other organisms
☒	☐ Clinical data
☒	☐ Dual use research of concern

Methods

n/a	Involved in the study
☒	☐ ChIP-seq
☒	☐ Flow cytometry
☒	☐ MRI-based neuroimaging

Antibodies

Antibodies used

Antibodies against proteins from baker's yeast Saccharomyces cerevisiae (are custom-made and are not commecially available) were generated in rabbits using peptides (Tim17, Tim21, Tim23, Tim44, Tim50, Mgr2, Aco1, Atp1, Cox1, Cox4, Cox12, Mdh1, Qcr8, Ssc1/mtHsp70, Tim22, Tom5, Tom22, Tom40, Tom70, Yme1). The antisera were used in 1:250-1,000 dilution. Primary anti-GFP Roche (#11814460001, clones 7.1 and 13.1, lot: 65309400) and secondary goat anti-rabbit-HRP Jackson ImmunoResearch Laboratories (#111-035-003, lot: 162282), goat anti-rabbit-HRP Sigma (A6154, lot: SLBG72001V), horse anti-mouse-HRP Cell Signaling Technology (#7076S, lot: 38) antibodies were obtained as listed. Secondary antibodies were used at concentrations of 1:5,000 to 1:20,000 (anti-rabbit HRP) or 1:2,000 (anti-mouse-HRP).

Validation

The specificity of the antibody raised against a protein from baker´s yeast (Saccharomyces cerevisiae) was controlled by comparing total cell extracts or mitochondrial lysates from wild-type yeast cells and the corresponding deletion strain or strains expressing a tagged version of the protein of interest via SDS-PAGE and Western blotting. Absence or size shift of the signal in cellular fractions of the mutant strain confirmed the specificity of the antibody signal. References for the antibodies are:
Rabbit polyclonal anti-Tim17, Ref. 65
Rabbit polyclonal anti-Tim21, Ref. 65
Rabbit polyclonal anti-Tim23, Ref. 65
Rabbit polyclonal anti-Tim44, Ref. 65
Rabbit polyclonal anti-Tim50, Ref. 65
Rabbit polyclonal anti-Mgr2, Ref. 65
Rabbit polyclonal anti-Aco1, Ref. 65
Rabbit polyclonal anti-Atp1, Ref. 66
Rabbit polyclonal anti-Cox1, Ref. 65
Rabbit polyclonal anti-Cox4, Ref. 65
Rabbit polyclonal anti-Cox12, Ref. 67
Rabbit polyclonal anti-Mdh1, Ref. 68
Rabbit polyclonal anti-Ssc1/mtHsp70, Ref. 67
Rabbit polyclonal anti-Qcr8, Ref. 65
Rabbit polyclonal anti-Tim22, Ref. 69
Rabbit polyclonal anti-Tom5, Ref. 70
Rabbit polyclonal anti-Tom22, Ref. 65
Rabbit polyclonal anti-Tom40, Ref. 65
Rabbit polyclonal anti-Tom70, Ref. 65
Rabbit polyclonal anti-Yme1, Ref. 65
Mouse monoclonal anti-GFP, Roche, 11814460001, manufacturer tested for functionality and purity relative to a reference standard to confirm the quality of each new reagent preparation, Gomkale et al., 2021, doi:10.1038/s41467-021-26016-1
